# The relationship among cognitive reserve and symptoms, cognition, and functioning in schizophrenia: A case–control study and meta-analysis

**DOI:** 10.1192/j.eurpsy.2026.12216

**Published:** 2026-05-12

**Authors:** Lingzhi Hou, Chuyuan Zhang, Haiyan Tang, Yinyu Peng, Peiyu Cao, Chongyang Han, Shuzhan Gao, Xijia Xu

**Affiliations:** 1 Department of Psychiatry, the Affiliated Brain Hospital of Nanjing Medical University, Nanjing, Jiangsu, China; 2 Department of Psychiatry, Nanjing Brain Hospital, Clinical Teaching Hospital of Medical School, Nanjing University, Nanjing, Jiangsu, China

**Keywords:** cognitive reserve, functional outcomes, negative symptoms, schizophrenia

## Abstract

**Background:**

Cognitive reserve (CR) is considered a positive factor in the onset, progression, and prognosis of diseases. It may also help explain the clinical heterogeneity observed in schizophrenia (SZ).

**Methods:**

This cross-sectional study included 70 patients with SZ and 64 healthy controls. Participants were assessed on CR, symptoms, cognition, and functional outcomes. Following PRISMA guidelines, we also searched PubMed, Scopus, Web of Science, Embase, the Cochrane Library, and PsycINFO for studies published up to September 25, 2025. Study quality was evaluated using the Newcastle-Ottawa Scale (NOS).

**Results:**

Patients with higher CR showed less severe negative and general psychopathological symptoms, along with better functional outcomes. Meta-analysis confirmed this relationship and further revealed positive correlations between CR and multiple cognitive domains, including speed of processing, working memory, verbal learning, visual learning, and reasoning and problem solving.

**Conclusions:**

This study demonstrates a positive association between CR and symptoms severity, cognitive performance, and functional outcomes in SZ.

## Introduction

Schizophrenia (SZ) is a neurodevelopmental brain disorder influenced by both genetic and environmental factors [1, 2]. Its lifetime prevalence is approximately 1% [3]. Most patients are difficult to diagnose early and treat effectively, placing a heavy burden on families and society. SZ is a complex psychiatric disorder characterized by a broad range of symptoms, including positive symptoms (hallucinations, delusions, disorganized speech and behavior), negative symptoms (deficits in motivation, pleasure, and expression), and cognitive impairments (deficits in attention, working memory, and executive function) [4]. Symptom progression is fluctuating, with the outcome often tending toward deterioration [5].

Cognitive reserve (CR) refers to the ability to optimize cognitive performance by recruiting alternative neural networks or making more efficient use of existing networks. This ability helps explain individual differences in susceptibility to the effects of brain aging, pathology, or damage on cognitive abilities or daily functioning [6]. SZ is a complex and severe mental disorder, and extensive research has consistently shown a poor correspondence between brain pathology and clinical symptoms in affected individuals [7], suggesting that other factors may influence this relationship. This incongruity underscores that, as brain development is shaped by both genetic and acquired influences, individuals could enhance the brain’s resilience to cope with aging and neuropathological damage through mechanisms involving neural plasticity and systemic regulation – for example, via education, cultural engagement, and functional training [8]. Within this context, Barnett et al. [9] proposed that CR may influence psychiatric disorders such as SZ by modulating the risk of onset and the expression of symptoms.

The commonly used proxies for CR include estimated premorbid intellectual quotient (IQ), educational level, occupational attainment, leisure activities, and premorbid adjustment [10–15]. Compared to single proxy indicators, the composite CR scores demonstrate greater sensitivity and construct validity, and better capture the multidimensional nature of CR [16]. Notably, the Hospital Clinic de Barcelona developed the Cognitive Reserve Assessment Scale in Health (CRASH) [17] in 2019, a scale specifically designed to assess CR in patients with severe psychiatric disorders. Its reliability and validity have been validated [17–19].

A recent study has shown that education, as an indicator of CR, significantly predicts the severity of negative symptoms in SZ [20], a finding consistent with prior research [21–24]. In addition, higher premorbid IQ appears to buffer the adverse effects of psychosis on cognition [25]. Previous research has estimated that each one-point decline below average premorbid IQ is associated with a 3.7% increase in schizophrenia risk [26]. Paula et al. [27] reported that individuals with higher CR may have a reduced risk of developing SZ and a later age of onset. Furthermore, these patients presented superior neuropsychological and social adaptation throughout the disease course. A systematic review [27] investigating the role of sociobehavioural indicators of CR in SZ found that a higher level of CR could delay the clinical diagnosis threshold and reduce symptom severity. Using principal component analysis, Amprodon-Boadas et al. [28] identified lower CR levels in children at familial high risk for schizophrenia during early development. This finding implies that cognitive reserve may act as a protective factor, potentially mitigating the risk of psychopathology and neurocognitive deficits. However, most investigations examining CR in SZ patients have relied on proxy measures, which limit comparability across studies.

The primary objective of this study was to explore the relationship between CR and clinical characteristics in patients with SZ, including symptoms, cognition, and functional outcomes. We used the CRASH scale to assess CR, as it allows for a more comprehensive evaluation of cognitive reserve capacity in all participants. We hypothesized that CR acts as a resilience factor, positively influencing the course of SZ – that is, individuals with higher CR would show less impairment across all measured domains. Additionally, although research on the role of CR in SZ exists, to the best of our knowledge, no systematic meta-analysis has yet summarized the evidence. Therefore, by integrating original case–control data with a meta-analysis, our approach provides more robust insights and an integrated perspective on these relationships.

## Method

### Case–control study

#### Study design and clinical assessment

This cross-sectional study included 70 inpatients with SZ (aged 18–55) from the Affiliated Brain Hospital of Nanjing Medical University between March and October 2025. Diagnoses were confirmed using a structured clinical interview according to the International Classification of Diseases, 10th revision (ICD-10). Patients with concomitant organic illnesses, traumatic brain injury, history of substance abuse, or serious behavioral disorders (e.g., aggressive behavior) were excluded. A total of 64 healthy controls, matched with the SZ group for sex, age, and education, were recruited through community advertisements. None of the controls had a personal or family history of psychiatric disorders, and the exclusion criteria were the same as those for the SZ group. General information was collected from all participants, including sex, age, BMI (body mass index, calculated as weight in kilograms divided by height in meters squared), educational level, marital status, and family history. Functional outcomes were assessed using the Functioning Assessment Short Test (FAST) [29], which evaluates six domains of functional impairment through 24 items, each scored from 0 to 3, with higher scores indicating greater impairment. The Positive and Negative Syndrome Scale (PANSS) [30] was used to assess symptom severity. It comprises positive, negative, and general psychopathology subscales, with supplementary items for aggression. Trained psychiatrists administered the PANSS through clinical examination and informant reports; higher total scores reflect greater illness severity. The study was approved by the Ethics Committee of Nanjing Brain Hospital (2017-KY017–02), and written informed consent was obtained from all participants.

#### Cognitive reserve and neuropsychological assessment

CR was assessed using the CRASH scale [17], which has demonstrated optimal psychometric properties and validity as a measure of CR [17–19]. The CRASH is a clinician-rated instrument, administered by trained clinical researchers through semi-structured clinical interviews combined with medical record information. It contains 33 items and assesses three domains considered fundamental to the CR construct: education, occupation, and intellectual and leisure activities. This last domain is evaluated across different life stages (childhood/adolescence, adulthood, and current situation). The scale takes approximately 15 minutes to administer, making it highly feasible. The maximum total score is 90, with higher scores indicating greater CR. Cognitive function was assessed in the SZ and HC groups using the MATRICS Consensus Cognitive Battery (MCCB). The assessment comprised nine tasks yielding raw scores (see Supplementary Material). Standardized scores were generated across seven cognitive domains using the MCCB computerized scoring program: speed of processing (SoP), attention/vigilance (AV), working memory (WM), verbal learning (Vrbl Lrng), visual learning (Vis Lrng), reasoning and problem solving (RPS), and social cognition (SC).

#### Statistical analysis

Statistical analyses were performed using SPSS 25.0 and GraphPad Prism 8.0. Normality of continuous variables was assessed using Q–Q plots and the Shapiro–Wilk test. Normally distributed data were presented as mean ± SD and compared using independent samples *t*-tests; Pearson correlation coefficients were computed to assess relationships between variables. Non-normally distributed data were expressed as median (interquartile range) and analyzed using non-parametric tests; Spearman’s rank correlation coefficients were used for correlation analysis. Categorical data were compared using chi-square tests. A two-tailed *p*-value <0.05 was considered statistically significant.

## Meta-analysis

We conducted a meta-analysis following the Preferred Reporting Items for Systematic Reviews and Meta-Analysis (PRISMA) guidelines. The protocol was registered in advance (PROSPERO: CRD420251183320).

### Literature search strategy

We systematically searched PubMed (Medline), Scopus, Web of Science, Embase, Cochrane Library, and PsycINFO on September 25, 2025. The search algorithm included MeSH terms and keywords adapted from the PubMed (MeSH Database), using the following strategy: (“Schizophrenia” OR Schizophrenias OR “Schizophrenic Disorders” OR “Disorder, Schizophrenic” OR “Disorders, Schizophrenic” OR “Schizophrenic Disorder” OR “Dementia Praecox”) AND (“Cognitive Reserve” OR “Cognitive Reserves” OR “Reserve, Cognitive” OR “Reserves, Cognitive” OR “Brain Reserve” OR “Brain Reserves” OR “Reserve, Brain” OR “Reserves, Brain”). Reference lists of included articles were hand-searched for additional studies. A snowballing approach [31] was also applied to identify further eligible studies from the reference lists of included articles. The initial literature search was conducted in November 2025. Prior to submission, we checked key databases (PubMed, Scopus, Web of Science) and confirmed that no additional eligible studies meeting our inclusion criteria had been published after the initial search date. Therefore, no updated search was performed.

### Study selection and quality assessment

**Figure 1.**
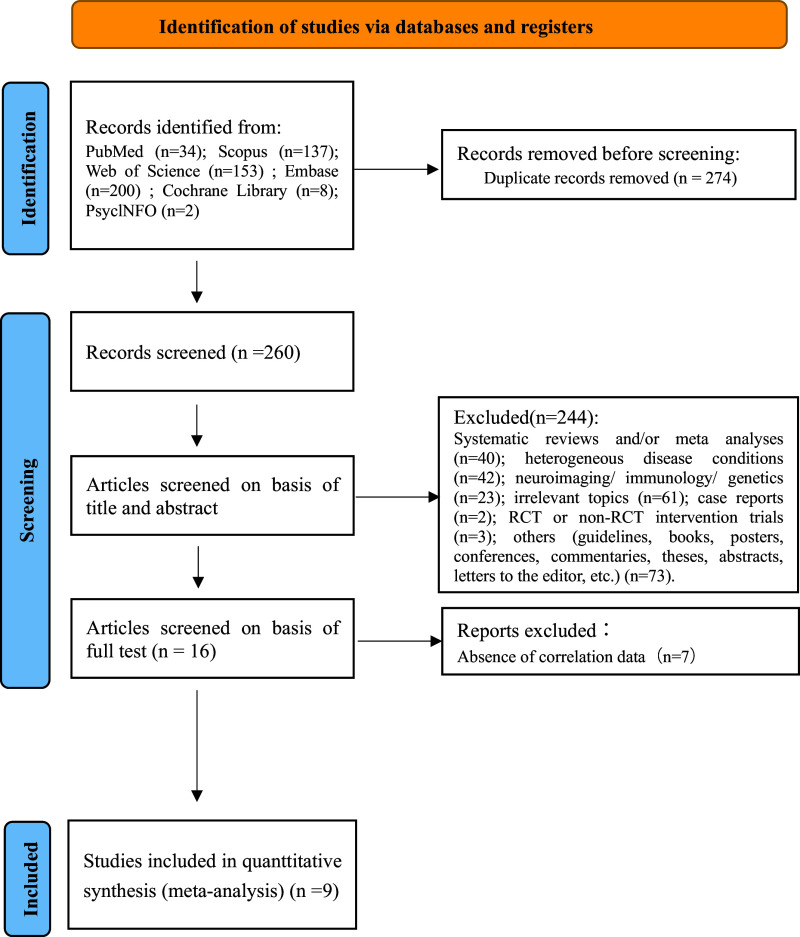
PRISMA flowchart of the literature search and study selection process. Figure 1. long description.

The inclusion criteria were: (i) studies reporting the effect of CR proxies on behavioral, cognitive, or functional outcomes in SZ patients; (ii) articles reporting quantitative research with a cross-sectional or longitudinal design (with only baseline measurements used). The exclusion criteria were: (i) systematic reviews, meta-analyses, case reports, case series, letters to editors or other non-peer-reviewed articles (e.g., books, book chapters, comments, theses, posters); (ii) articles based on neuroimaging, immunology, genetics studies that did not report behavioral data; (iii) studies not focusing on individuals diagnosed with SZ; (iv) papers not written in English. Following PRISMA guidelines [32], a flowchart of the selection process is presented in Figure 1.

A quality assessment of the included studies was conducted using a modified version of the Newcastle-Ottawa Scale (NOS) [33]. This scale comprises three sections evaluating three quality parameters (selection, comparability, and outcome), with seven specific items (see Supplementary Material). Scores range from 0 to 10, with higher scores indicating better study quality. Scores of 0–4 were considered “unsatisfactory,” 5–6 “satisfactory,” 7–8 “good,” and 9–10 “very good.” As in the study selection phase, two independent reviewers, Lingzhi Hou and Chuyuan Zhang, conducted the quality assessments, and a third reviewer, Xijia Xu, was consulted if necessary.

### Statistical analysis

Statistical analyses were conducted using STATA 17.0, with statistical significance set at *p* < 0.05. For each included study, the correlation coefficient (*r*) between cognitive reserve and outcome variables, along with the sample size (*n*), was extracted. Each coefficient was transformed to Fisher’s *Z* to approximate a normal distribution. A random-effects model was then used to pool the weighted Fisher’s *Z* values across studies; these were back-transformed after pooling to obtain overall r values with their 95% confidence intervals (CIs) and *p* values, following previous research [33–35]. Correlation strength was classified as weak (0–0.3), moderate (0.3–0.7), or strong (>0.7) based on standard cutoffs [36]. The Cochran’s *Q* test (*p* < 0.10 indicative) [37], and the *I* ^2^ statistic (>50% indicative) [38] were used to explore heterogeneity. When high heterogeneity was identified, meta-regressions were conducted on the following variables: year of publication, age, sex, PANSS scores, and age at onset. For associations including at least ten studies, publication bias was assessed by visual inspection of funnel plots and the Egger’s test [39]. Leave-one-out sensitivity analysis was performed to verify the robustness of the associations.

## Results

### Case–control study

#### Sociodemographic characteristics of the sample

Table 1 shows the sociodemographic and clinical characteristics of the participants. The mean ages of the SZ and HC groups were 29.84 ± 7.33 and 27.98 ± 6.73, with female proportions of 55.71 and 54.69%, respectively. There were no significant differences between SZ and HC groups in sex, age, BMI, years of education, or marital status (all *p* > 0.05).

**Table 1.** Demographic and clinical characteristics of SZ and HC groups Table 1. long description.

	SZ (*N* = 70)	HC (*N* = 64)	Statistics	*p*-value	Effect sizes
Sex			0.014	0.905	0.010
Female, *n* (%)	39 (55.71%)	35 (54.69%)			
Male, *n* (%)	31 (44.29%)	29 (45.31%)			
Age (years)	29.84 ± 7.33	27.98 ± 6.73	1.524	0.130	0.264
BMI	22.92 (19.88 ~ 25.41)	22.35 (20.50 ~ 24.53)	−0.624	0.533	−0.570
Education (years)	14.19 ± 3.22	15.09 ± 2.92	−1.703	0.091	−0.295
Marital status			1.196	0.274	0.094
Unmarried, *n* (%)	53 (75.71%)	43 (67.19%)			
Married, *n* (%)	17 (24.29%)	21 (32.81%)			
Family history of psychosis			17.801	<0.001	0.364
Yes, *n* (%)	17 (24.29%)	0 (0.00%)			
No, *n* (%)	53 (75.71%)	64 (100.00%)			
Prior psychiatric medication use			26.730	<0.001	0.447
Yes, *n* (%)	24 (34.29%)	0 (0.00%)			
No, *n* (%)	46 (65.71%)	64 (100.00%)			
Duration of illness (years)	4.87 ± 4.88				
Length of stay	20.56 ± 9.37				

#### Comparison of the differences between SZ and HC groups

Patients with SZ scored 86.41 ± 10.83 on PANSS total scale and 32.34 ± 9.28 on CRASH scale. The CR score differed significantly between SZ and HC groups (*p* < 0.001). Except for working memory (*p* = 0.261), patients demonstrated significant impairments across all cognitive domains – including speed of processing, attention/vigilance, verbal learning, visual learning, reasoning and problem solving, and social cognition – compared to HC group (*p* < 0.001). Patients also exhibited higher FAST scores relative to healthy participants (*p* < 0.001) (Table 2).

**Table 2.** Differences in clinical, cognitive and functional characteristics for SZ and HC groups Table 2. long description.

	SZ (*N* = 70)	HC (*N* = 64)	Statistics	*p*-value	Effect sizes
PANSS total score	86.41 ± 10.83				
PANSS-P	23.54 ± 5.24				
PANSS-N	19.73 ± 6.19				
PANSS-G	43.11 ± 5.96				
Overall composite	29.39 ± 10.75	43.56 ± 9.45	−8.073	<0.001	−1.396
Speed of processing	35.93 ± 11.68	49.16 ± 10.43	−6.89	<0.001	−1.192
Attention/vigilance	36.24 ± 10.48	46.91 ± 9.08	−6.269	<0.001	−1.084
Working memory	38.46 ± 14.93	40.84 ± 9.05	−1.129	0.261	−0.191
Verbal learning	39.89 ± 12.84	46.97 ± 9.27	−3.683	<0.001	−0.628
Visual learning	40.01 ± 10.98	50.34 ± 6.82	−6.599	<0.001	−1.119
Reasoning and problem solving	39.99 ± 11.33	51.88 ± 8.54	−6.895	<0.001	−1.178
Social cognition	30.46 ± 8.08	35.91 ± 10.61	−3.361	0.001	−0.581
FAST	46 (38.00 ~ 55.00)	4 (2.00 ~ 6.00)	−9.993	<0.001	−0.860
CRASH	32.34 ± 9.28	41.85 ± 9.81	−5.764	<0.001	−0.997

*Note:* CRASH, the cognitive reserve assessment scale in health; FAST, the functioning assessment short test; PANSS, the positive and negative syndrome scale; PANSS-G, general psychopathology symptom in PANSS scale; PANSS-P, positive symptom in PANSS; PANSS-N, negative symptom in PANSS scale.

#### Correlation analyses between SZ and HC groups

**Figure 2.**
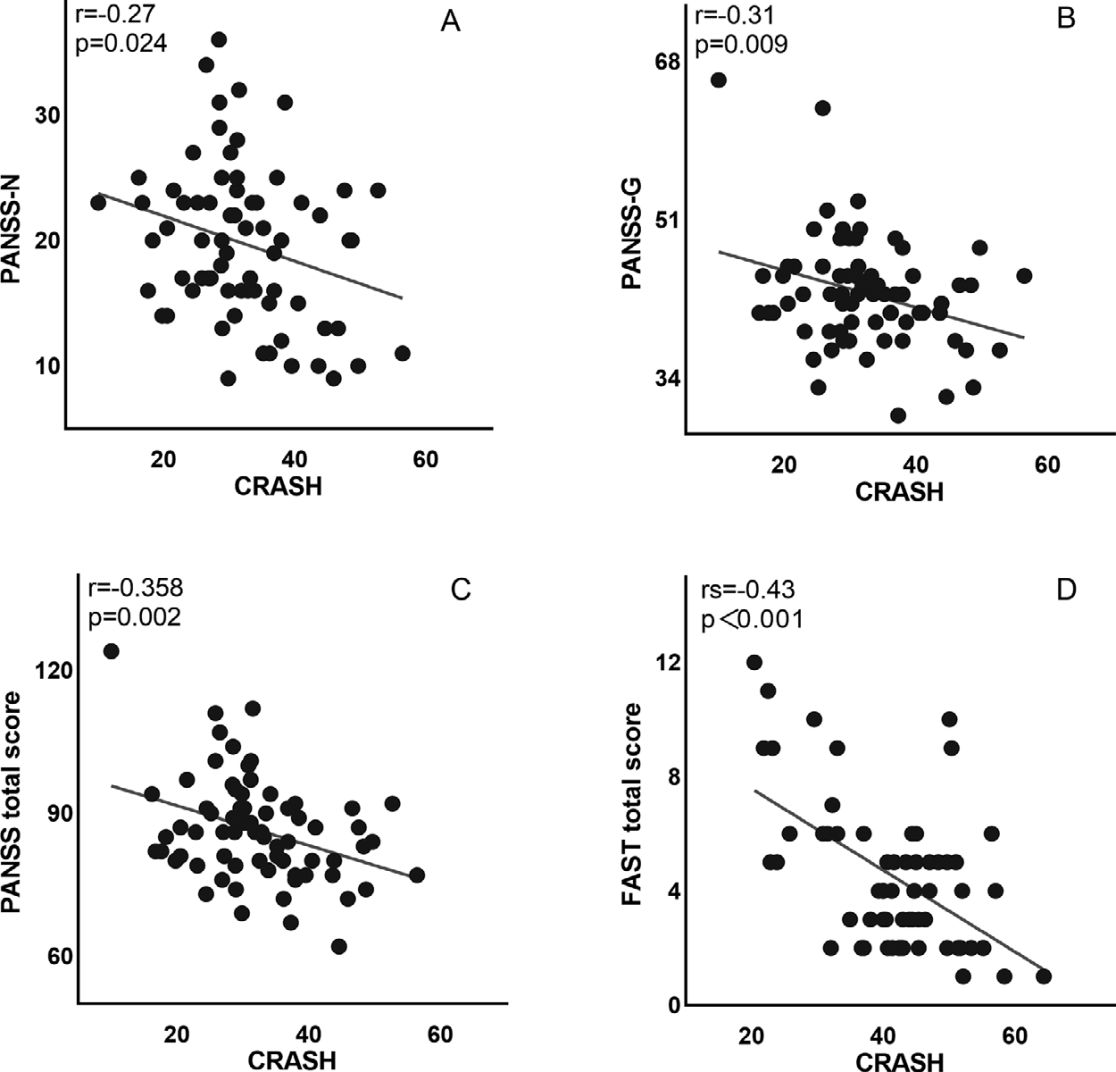
Association between CR and symptoms and functional outcomes in the SC group. *Note:* CRASH, the cognitive reserve assessment scale in health; FAST, the functioning assessment short test; PANSS, the positive and negative syndrome scale; PANSS-P, positive symptom in PANSS; PANSS-N, negative symptom in PANSS scale; PANSS-G, general psychopathology symptom in PANSS scale. Figure 2. long description.

In the SZ group, CRASH score was negatively correlated with clinical symptoms – specifically negative symptoms (*r* = −0.27), general symptoms (*r* = −0.31), and PANSS total score (*r* = −0.358) – and, most strongly, with functional impairment as measured by the FAST (*r* = −0.547) (Figure 2). No significant correlations were found between CR and age, illness duration, or cognitive performance (all *p* > 0.05) (see Supplementary Material). In HC group, the CRASH score was negatively correlated with the FAST score (*r* = −0.540, *p* < 0.001), but no significant correlations were observed between the CRASH score and any cognitive domains (all *p* > 0.05) (see Supplementary Material).

### Meta-analyses

#### Study selection

The study selection process is shown in the PRISMA flowchart (Figure 1). A total of 532 articles were identified through a systematic search of electronic databases. After removing 274 duplicates, 260 articles underwent title and abstract screening. Following the exclusion of 244 irrelevant articles, 16 full-text reports were assessed, and 7 were excluded.

Thus, nine studies [20, 24, 40–46] were included in the meta-analysis. Together with our case–control study, a total of 10 studies comprising 1789 patients with SZ were analyzed. Notably, in two of the included studies [40, 46], participants were diagnosed with schizophrenia spectrum disorders. Given that patients with schizophrenia accounted for the highest proportion in these two studies [40, 46], both were nevertheless included in the meta-analysis.

#### Characteristics of included studies

Table 3 summarizes the characteristics of the 10 included studies. They were published between 2009 and 2025 and included a total of 1789 individuals with SZ. Females comprised 36% of the proportion, and the mean age was 31.64 ± 10.6 years. Six studies were cross-sectional, and four were longitudinal. Quality assessment showed that one study was of very good quality (scoring 9), eight were of good quality (scoring 7 or 8), and one was of satisfactory quality (scoring 5 or 6). No studies were rated as unsatisfactory. Detailed quality assessment results are provided in the SupplementarySupplementary Material.

**Table 3.** Main findings of studies investigating the relationship among CR and symptoms, cognition, and functioning in SZ Table 3. long description.

Author (year)	*N*	Age (mean ± SD)	Diagnostic instrument	Measure of CR	Measure of symptoms	Measure of cognitive function	Measure of functioning	Main results	Quality of the study (NOS)
Present study (2025)	70	29.84 ± 7.33	ICD 10	CRASH	PANSS	SoP: TMT-A, BACS, CF; AV: CPT-IP, WM: WMS-III SS, Vrbl Lrng: HVLT-R, Vis Lrng: BVMT-R, RPS: NAB Mazes; SC: MSCEIT	FAST	A statistically significant relationship between CR and PANSS and FAST was found with higher CR related to better performance in clinical symptoms and social functioning.	8 Good
Leeson et al. (2009) [40]	129	25.53 ± 8.04	DSM-IIIR ICD 10	Premorbid IQ	PANSS	WM: Spatial working memory task; Vrbl Lrng: RAVLT; RPS: Tower of London task	NA	Both the low and the deteriorated groups had more core negative symptoms, verbal learning, working memory and planning were correlated with premorbid IQ at P < .05.	7 Good
Cámara et al. (2021) [41]	116	40.66 ± 9.62	ICD 10	Education	PANSS	SoP: Symbol search; WM: Digit Span; RPS: Phonological verbal fluency	GAF	Education showed significant positive correlations with working memory and verbal fluency tasks, education was negatively correlated with GAF.	8 Good
Correa-Ghisays et al. (2022) [42]	30	40.8 ± 10.7	DSM-V	Vocabulary, Education	PANSS	SoP: FTT, WAIS-III Digit Symbol Coding subtest, SCWT Color and Word subtests, TMT-A; WM: TMT-B, WAIS-III Digit Span-B subtest; Vrbl Lrng: TAVEC V3, V8 and V10; Vis Lrng: ROCF; RPS: SCWT Color/Word subtest, WCST-CCPE	FAST	CR was negatively correlated with clinical symptoms and positively correlated with cognitive and social functioning.	7 good
Ehrminger et al. (2020) [43]	776	31.6 ± 9.3	DSM-IV	Premorbid IQ, Education	PANSS	NA	GAF	CR scores were associated with GAF scores and cognitive scores.	6 Satisfactory
Rodriguez et al. (2022) [44]	137	25.93 ± 6.47	ICD-10	Education, Premorbid IQ, Socioeconomic status	PANSS	NA	GAF	CR was positively to symptom severity and general functioning.	8 Good
Sánchez-Torres et al. (2023) [45]	99	26.25 ± 5.78	DSM-IV	Premorbid IQ, Education, Scholastic performance	PANSS	SoP: TMT-A; AV: CPT-II; WM: Tower of London, WAIS-III Digit SpanTest; Vrbl Lrng: AIS-III Letter-Number Sequencing, TAVEC; Vis Lrng: WMS-Visual reproduction subtest; SC: MSCEIT	FAST, GAF	CR is associated with cognitive performance in processing speed and working memory and visual memory and social cognition.	7 Good
Amoretti et al. (2024) [24]	225	45.02 ± 12.82	DSM-V	CRASH	PANSS	SoP: PST; Vrbl Lrng: VLT-D	GAF	CR predicted all cognitive domains, negative symptoms and functioning	9 Very Good
Forte et al. (2024) [46]	138	24.77 ± 5.29	DSM-IV	Premorbid IQ, Education, Lifetime participation in leisure, social, Physical activities	PANSS	NA	FAST	CR is negatively correlated with clinical symptoms.	7 Good
Spinelli et al. (2025) [20]	69	39.23 ± 13.85	NA	Education	PANSS	SC: Reading the Mind in the Eyes Test	NA	CR plays a significant role in alleviating negative symptoms in patients with schizophrenia.	7 good

*Note:* AV, attention/vigilance; BACS, brief assessment of cognition in schizophrenia; BVMT-R, brief visuospatial memory test revised; CR, cognitive reserve; CRASH, Cognitive Reserve Assessment Scale in health; CPT-IP, continuous performance test-identical paris; CF, category fluency; CPT-II, Continuous Performance Test-II; DSM-IV, Diagnostic and Statistical Manual of Mental Disorders, 4 Edition; DSM-V, Diagnostic and Statistical Manual of Mental Disorders, 5 Edition; DSM-IIIR, Diagnostic and Statistical Manual of Mental Disorders, 3 Edition; FTT, Finger Tapping Test; FAST, Functioning Assessment Short Test; GAF, Global Assessment of Functioning; HVLT-R, Hopkins Verbal Learning Test-Revised; HT, Hinting Task; IQ, intelligence quotient; ICD-10, International Classification of Diseases, 10 revision; MSCEIT, Mayer-Salovey-Caruso Emotion Intelligence Test; NAB Mazes, Neuropsychological Assessment Battery:mazes; PANSS, Positive and Negative Syndrome Scale; PST, processing speed test; RAVLT, Rey Auditory Verbal Learning Task; RMET, Reading the Mind in the Eyes Test; RPS, reasoning and problem solving; SC, social cognition; SCWT, Stroop Color and Word Test; TAVEC, Complutense Verbal Learning Test; TMT-A, Trail Making Test A; VLT-D, Verbal Learning Test-Delayed; Vis Lrng, visual learning; Vrbl Lrng, verbal learning; WAIS-III, Wechsler Adult Intelligence Scale 3rd Version; WCST-128, Wisconsin Card Sorting Test; WCST, Wisconsin Card Sorting Test; WM, working memory; WMS-III SS, Wechsler Memory Scale-Third Edition: spatial span.

#### Main analyses

**Figure 3.**
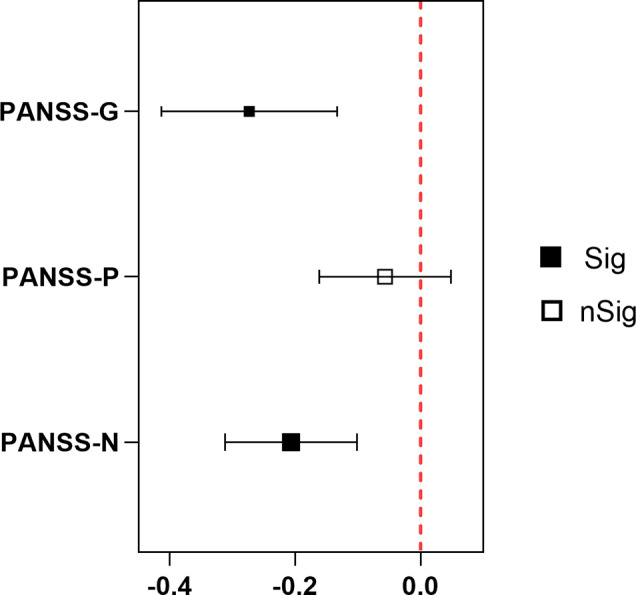
Overall results of the meta-analysis combining CR and PANSS. *Note:* PANSS, the positive and negative syndrome scale; PANSS-P, positive symptom in PANSS; PANSS-N, negative symptom in PANSS scale; PANSS-G, general psychopathology symptom in PANSS scale. The *x*-axis denotes the correlation coefficient (*r*) and horizontal error bars indicate 95% confidence intervals.

The main results of the meta-analyses examining the relationship between CR and PANSS domains are presented in Table 4 and Figure 3. Overall, higher levels of CR were significantly associated with less severe negative symptoms (*r* = −0.21, *p* < 0.001) and less severe general psychopathology symptoms (*r* = −0.28, *p* < 0.001) in patients with SZ. No consistent correlation was found between CR and positive symptoms. Heterogeneity was relatively high for the association with negative symptoms (*I^2^* = 60.93, *Q*-test *p*-value = 0.01). Forest plots for individual study results are available in the Supplementary Material.

**Table 4.** Results of the meta-analysis exploring the relation between CR and PANSS domains Table 4. long description.

PANSS	Studies, *n*	Patients, *n*	Overall effect size, r	95% CI	*r p-*value	*I^2^* (%)	*Q*-test (df)	*Q*-test*p*-value
PANSS-N	8	914	−0.21	−0.31, −0.10	<0.001	60.93	17.89	0.01
PANSS-P	5	523	−0.06	−0.16, 0.05	0.270	23	5.24	0.26
PANSS-G	3	169	−0.28	−0.41, −0.13	<0.001	0.00	1.27	0.53

*Note*: PANSS, The Positive and Negative Syndrome Scale; PANSS-P, positive symptom in PANSS; PANSS-N, Negative Symptom in PANSS Scale; PANSS-G, General Psychopathology Symptom in PANSS Scale.

**Figure 4.**
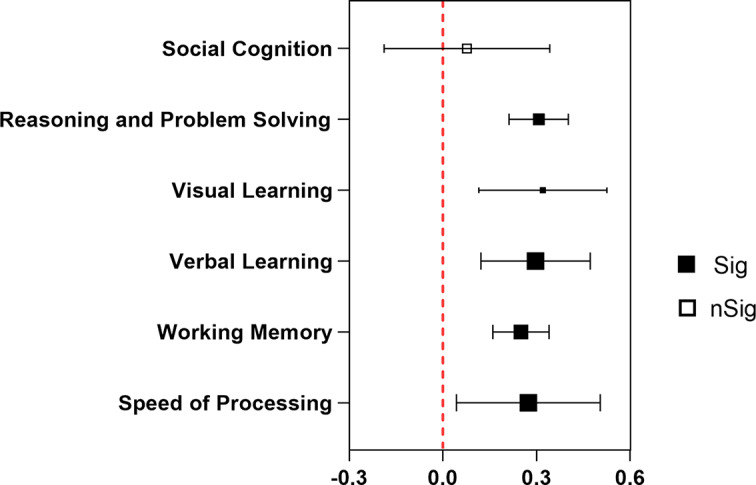
Overall results of the meta-analysis combining CR and cognition domains. *Note:* The *x*-axis denotes the correlation coefficient (*r*) and horizontal error bars indicate 95% confidence intervals. Figure 4. long description.

The main results of the meta-analyses examining the relationship between CR and cognitive domains are presented in Table 5 and Figure 4. Significant positive correlations were found between CR and multiple cognitive domains: speed of processing (*r* = 0.28), working memory (*r* = 0.25), verbal learning (*r* = 0.30), visual learning (*r* = 0.33), and reasoning and problem solving (*r* = 0.31). Heterogeneity was high for all cognitive domains except working memory (*I^2^* = 0.00, *Q*-test *p*-value = 0.93) and reasoning and problem solving (*I^2^* = 0.00, Q-test *p*-value = 0.56). Forest plots are provided in the Supplementary Material.

**Table 5.** Results of the meta-analysis exploring the relation between CR and cognitive domains Table 5. long description.

Cognition	Studies, *n*	Patients, *n*	Overall effect size, *r*	95% CI	*r p*-value	*I* ^2^(%)	*Q*-test (df)	*Q*-test*p*-value
Speed of processing	5	540	0.28	0.04, 0.50	0.020	86.87	42.16	<0.001
Working memory	5	444	0.25	0.16, 0.34	<0.001	0.00	0.84	0.930
Verbal learning	5	553	0.30	0.12, 0.47	0.002	77.90	17.42	0.002
Visual learning	3	199	0.33	0.11, 0.52	0.004	57.18	4.82	0.090
Reasoning and problem solving	4	345	0.31	0.21, 0.40	<0.001	0.00	2.08	0.560
Social cognition	3	238	0.08	−0.19, 0.34	0.560	76.27	8.34	0.020

**Figure 5.**
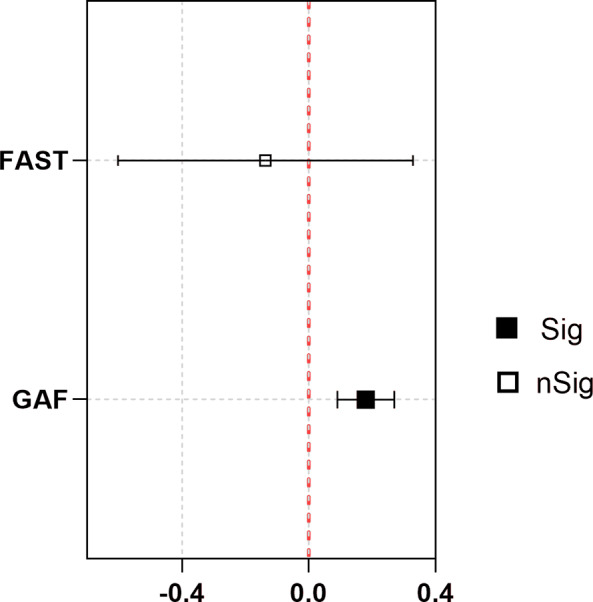
Overall results of the meta-analysis combining CR and functional outcomes. *Note:* FAST, the functioning assessment short test; GAF, the global assessment of functioning. The *x*-axis denotes the correlation coefficient (*r*) and horizontal error bars indicate 95% confidence intervals. Figure 5. long description.

The main results of the meta-analyses examining the relationship between CR and functional outcomes are presented in Table 6 and Figure 5. A weak positive correlation was found between CR and GAF scores (*r* = 0.18), while no significant association was observed between CR and FAST scores (*p* > 0.05). Considerable heterogeneity was present for the FAST analysis (*I* ^2^ = 92.21, *Q*-test *p* < 0.001). Forest plots are available in the Supplementary Material.

**Table 6.** Results of the meta-analysis exploring the relation between CR and functional outcomes Table 6. long description.

Social function	Studies, *n*	Patients, *n*	Overall effect size, *r*	95% CI	*r p*-value	*I* ^2^(%)	*Q*-test (df)	*Q*-test *p* value
GAF	4	1254	0.18	0.09, 0.27	<0.001	50.21	6.08	0.110
FAST	3	238	−0.16	−0.59, 0.34	0.55	92.21	19.37	<0.001

*Note*: FAST, the Functioning Assessment Short Test; GAF, the global assessment of functioning.

#### Sensitivity analysis and meta-regression analyses

Leave-one-out sensitivity analyses were conducted to assess the robustness of the findings. The following associations changed significance after removing individual studies: (i) the correlation for processing speed became non-significant after removing the study by Correa-Ghisays et al. [42]; (ii) the correlation for social cognition became non-significant after removing our case–control study; (iii) results for functional outcomes (FAST) were highly inconsistent, with effect sizes varying and confidence intervals crossing zero.

Meta-regression analyses were performed to explore potential moderators of the observed relationships. Lower PANSS scores significantly predicted stronger correlations between CR and visual learning and between CR and social cognition. A higher proportion of females significantly predicted a stronger correlation between CR and functional outcomes measured by GAF, whereas a lower proportion of females significantly predicted a stronger correlation between CR and functional outcomes measured by FAST. Other variables examined were not significant. Due to the limited number of studies (fewer than ten) for each association, publication bias could not be assessed. Detailed results of sensitivity analyses and meta-regressions are provided in the Supplementary Material.

## Discussion

This study, combining original clinical data with a meta-analytic approach, suggests that higher CR may be associated with better clinical, cognitive, and functional outcomes in patients with SZ. As the first meta-analytic investigation of CR in individuals with SZ, these findings highlight the potential protective role of CR while also underscoring the preliminary nature of the current evidence.

In our cohort and meta-analyses, higher CR was associated with less severe negative symptoms, consistent with previous research [10, 20, 24, 40–42, 44]. Negative symptoms are a core feature of SZ, and high levels of CR proxies have repeatedly been linked to less severe psychotic symptoms [27]. For instance, in a study of 69 individuals with schizophrenia spectrum disorders (SSD), educational attainment emerged as a significant predictor of negative symptom severity [20]. Leeson et al. [40], who classified 129 SZ patients by current and premorbid IQ profiles, found that both low-IQ and deteriorated-IQ subgroups had longer index admissions and more severe core negative symptoms at three-year follow-up. Rodriguez et al. [44], using education, premorbid IQ, and socioeconomic status as CR proxies in SSD, also revealed that patients with lower CR exhibited more severe negative symptoms. Amoretti et al. [24] employed the CRASH scale to assess CR, and their findings suggested that the implications of CR depend on illness stage (chronic versus early), with a stronger effect on negative symptoms in chronic SZ.

In contrast, we found no association between CR and positive symptoms in patients with SZ, a finding that is supported by most existing studies [20, 24, 40, 42]. Although both positive and negative symptoms are core features of SZ, negative symptoms are closely associated with structural and homeostatic brain resources and represent deficits related to neurodevelopmental abnormalities [47] – precisely the fundamental neurobiological basis of CR [8]. CR, as a form of neuroplastic capacity accumulated through factors such as education and occupational achievement, is thought to counteract functional deficits resulting from structural damage by recruiting alternative neural networks or enhancing the efficiency of existing ones [6], thereby alleviating negative symptoms such as apathy and social withdrawal [24, 40]. Positive symptoms, in contrast, are more closely linked to functional disturbances in specific neural circuits, particularly hyperdopaminergia in the mesolimbic pathway, and are characterized by acute onset and fluctuation [48]. CR, as a relatively static or slowly modulating structural resource, may mitigate chronic, progressive structural decline but cannot quickly counteract acute chemical imbalances triggered by surges in neurotransmitter activity. This may partly explain the lack of a direct, linear association between CR and positive symptoms severity.

Additionally, this study identified associations between CR and the PANSS general psychopathology subscale [20, 42, 44]. However, this subscale reflects the breadth and overall severity of symptom presentation, rendering it less valid as a specific construct compared to the more narrowly defined negative and positive symptom subscales. In general, CR appears to attenuate the clinical manifestation of SZ, with patients exhibiting higher CR showing better trajectories in illness symptom development.

In our case–control study, no significant correlations were observed between CR and cognitive domains in patients with SZ – a finding that diverges from the meta-analysis results. CR is defined as the ability to optimize or maximize performance through differential recruitment of brain networks [6]. That is, at the same level of clinical severity, individuals with higher CR may tolerate more severe brain pathology [6, 49]. This leads to a plausible hypothesis: among SZ patients with similar levels of cognitive impairment, those with higher CR may harbor more severe neuropathological changes. Therefore, investigating the complex interplay between brain damage, clinical presentation, and the mediating effect of CR in SZ is likely to provide important insights. On the other hand, limited sample size and differences in measurement instruments may partly account for the inconsistent findings.

Previous studies have suggested that high premorbid IQ may buffer the adverse effects of psychosis on cognition [25, 40, 50]. Similarly, Holthausen and colleagues [51] observed that patients who maintained normal cognitive performance had significantly higher IQ and education compared to those with cognitive impairment, suggesting that differences in cognitive compensation capacity may explain the existence of cognitively impaired subgroups. Among chronic SZ patients, higher educational attainment may ameliorate executive dysfunction [41], and social cognition may moderate the predictive effect of education on negative symptoms [20]. Furthermore, in patients carrying cognitive impairment risk alleles (ACE D and APOE-ε4), elevated education appears to mitigate the impact of schizophrenia-related genetic polymorphisms on cognitive performance [52]. Beyond these measures, premorbid adjustment has also been utilized as a proxy for CR. Adolescent premorbid adjustment significantly predicts cognitive function (speed of processing, executive function, working memory, verbal memory, and social cognition) in SZ patients [53]. In clinically stabilized outpatients with SZ, Buonocore et al. [15] found significant correlations between premorbid adjustment and working memory, with both premorbid adjustment and age serving as significant predictors of overall cognitive improvement after cognitive remediation therapy. Among patients who did not relapse over a three-year follow-up, those with higher CR (based on premorbid IQ, education, and scholastic performance) demonstrated better speed of processing and visual memory than those who experienced relapse [45]. Another longitudinal study revealed that CR partially mediated the relationship among baseline attention, verbal memory, and working memory and functional outcomes at follow-up [54]. Examining CR from a gender perspective using proxies of premorbid IQ, years of education, and employment status in first-episode psychosis (FEP), Herrero et al. [55] found that female patients had higher CR than males. In the low-CR subgroup, females showed superior verbal memory and processing speed, while among high-CR patients, males exhibited better attentional performance. Although our meta-analysis highlighted a positive effect of CR on several cognitive domains in SZ, significant heterogeneity was observed across studies. Consequently, these findings have limited generalizability, underscoring the necessity for further primary research in this area.

Our case–control study also indicates that SZ patients with higher CR exhibit better functional outcomes (FAST) compared to those with lower CR, consistent with previous research. Holthausen et al. [51], employing the Groningen Social Disabilities Schedule (GSDS), observed that higher IQ was correlated with greater overall functional outcomes in individuals with SZ. Similarly, using education as a proxy for CR, Cámara et al. [41] reported a significant association between higher educational attainment and fewer daily behavioral problems, as well as better functional performance (GAF). This finding is further supported by Rodriguez and colleagues [44]. A ten-year longitudinal study that classified first-episode non-affective psychosis patients into five CR-based clusters – defined by premorbid IQ, years of education, and premorbid adjustment – revealed that the cluster with the most deficient CR (C1) also showed the most impaired social functioning (DAS), in contrast to the higher CR clusters (C4 and C5) [56]. However, a recent study found that the implications of CR depend on the stage of the disease: it predicts functional outcomes (GAF) in chronic SZ but not in early-stage SZ [24]. Of note, despite variations in functional assessment tools across studies, most evidence confirms that SZ with higher CR consistently show stronger functional outcomes, both cross-sectionally and longitudinally. This consensus aligns with our meta-analysis on the CR and GAF relationship and a systematic review by Herrero et al [27]. Our meta-analysis also integrated three studies examining the CR–FAST relationship in SZ. While our case–control results concur with those reported by Forte et al. [46], they diverge from the findings of Patricia and colleagues [42], who identified a positive CR–FAST correlation in a transdiagnostic sample including 30 SZ patients. The small sample size may explain this discrepancy. Moreover, the meta-analysis itself, based on only three studies, yielded a highly unstable pooled estimate.

Notably, the high heterogeneity observed in this meta-analysis may be partly attributable to the relatively small sample sizes of the included studies. However, high heterogeneity is not a limitation unique to this study, but rather a common phenomenon in this field. Even in meta-analyses with a relatively large number of included studies (outside the field of psychiatry), heterogeneity remained significantly high [12, 57, 58]. As a complex and multidimensional construct, CR currently lacks unified operational criteria. Variations across studies in the selection of measurement tools and inclusion criteria contribute to considerable freedom in study design, limiting the comparability of results. This variability in measurement and operationalization may be the underlying reason for the persistently high heterogeneity observed in meta-analyses within this field. As the first meta-analysis to investigate the characteristics of CR in patients with SZ, the core value of this study lies not in providing a single, precise pooled effect size, but in systematically revealing and quantifying the extent of heterogeneity present in current research. Concurrently, this study provides crucial empirical evidence for future research to delve into the sources of heterogeneity through methods such as subgroup analyses and meta-regression.

This study has several limitations. First, the sample size of patients was relatively small (*N* = 70) with an uneven age distribution: 58 patients (82.9%) were aged 18–35 years, and only 12 (17.1%) were aged 36–55 years. Most patients (66%) were first-episode and medication-naïve with short illness duration. This restricted variance in age and illness duration may explain the null associations with CR. Second, although the CRASH scale comprehensively assesses education, occupation, and lifelong activities, it does not include IQ – a common proxy for CR. Future studies should consider supplementing the CRASH with the WAIS-IV vocabulary subtest to assess premorbid IQ. Third, variability in the measurement tools for CR limits the interpretability of pooled results. Additionally, the diversity of neuropsychological tests poses methodological challenges, as our categorization of cognitive domains relied on the original authors’ classifications, potentially introducing bias.

## Conclusion

Higher CR appears to positively moderate the impact of pathology on clinical course, functional outcomes, and cognitive performance in patients with SZ. Given that early identification of patients at risk for poor prognosis remains a major clinical challenge, assessing CR may help identify individuals more susceptible to unfavorable outcomes and inform therapeutic strategies to promote recovery.

## Supporting information

10.1192/j.eurpsy.2026.12216.sm001 Hou et al. supplementary material Hou et al. supplementary material

## Data Availability

Data are available upon request from the corresponding author.

## Supplementary material

The supplementary material for this article can be found at http://doi.org/10.1192/j.eurpsy.2026.12216.

## Figure 1. Long description

At the top, a yellow box states Identification of studies via databases and registers. Below, the first box lists Records identified from PubMed 34, Scopus 137, Web of Science 153, Embase 200, Cochrane Library 8, PsycINFO 2. An arrow right leads to Records removed before screening: Duplicate records removed 274. The main vertical path continues to Records screened 260. An arrow right leads to Excluded 244, with breakdown: Systematic reviews and or meta analyses 40, heterogeneous disease conditions 42, neuroimaging or immunology or genetics 23, irrelevant topics 61, case reports 2, RCT or non-RCT intervention trials 3, others (guidelines, books, posters, conferences, commentaries, theses, abstracts, letters to the editor, etc.) 73. The main path continues to Articles screened on basis of title and abstract, then to Articles screened on basis of full text 16. An arrow right leads to Reports excluded: Absence of correlation data 7. The final box at the bottom states Studies included in quantitative synthesis (meta-analysis) 9. The left margin labels each stage: Identification, Screening, Included.

Navigate back to Figure 1..

## [Table 1] Long description

Beginning at the top, the table lists variables in the leftmost column: sex, age, B M I, education, marital status, family history of psychosis, prior psychiatric medication use, duration of illness, and length of stay. For sex, S Z group has 39 females (55.71 percent) and 31 males (44.29 percent); H C group has 35 females (54.69 percent) and 29 males (45.31 percent). Age is 29.84 plus or minus 7.33 years for S Z and 27.98 plus or minus 6.73 years for H C. B M I is 22.92 (range 19.88 to 25.41) for S Z and 22.35 (range 20.50 to 24.53) for H C. Education is 14.19 plus or minus 3.22 years for S Z and 15.09 plus or minus 2.92 years for H C. Marital status shows S Z group with 53 unmarried (75.71 percent) and 17 married (24.29 percent); H C group with 43 unmarried (67.19 percent) and 21 married (32.81 percent). Family history of psychosis is present in 17 S Z participants (24.29 percent) and absent in all H C participants. Prior psychiatric medication use is reported in 24 S Z participants (34.29 percent) and none in H C. Duration of illness for S Z is 4.87 plus or minus 4.88 years; length of stay is 20.56 plus or minus 9.37. Statistical values, p-values, and effect sizes are provided for each variable, with significant differences in family history and medication use (p less than 0.001).

Navigate back to [Table 1].

## [Table 2] Long description

Starting from the top row, the table lists measures in the leftmost column: PANSS total score, PANSS-P, PANSS-N, PANSS-G, overall composite, speed of processing, attention/vigilance, working memory, verbal learning, visual learning, reasoning and problem solving, social cognition, FAST, and CRASH. For S Z (N equals 70), PANSS total score is 86.41 plus or minus 10.83, PANSS-P is 23.54 plus or minus 5.24, PANSS-N is 19.73 plus or minus 6.19, PANSS-G is 43.11 plus or minus 5.96. H C (N equals 64) values are not listed for PANSS scores. For overall composite, S Z is 29.39 plus or minus 10.75, H C is 43.56 plus or minus 9.45, with statistics negative 8.073, p value less than 0.001, effect size negative 1.396. Speed of processing: S Z 35.93 plus or minus 11.68, H C 49.16 plus or minus 10.43, statistics negative 6.89, p value less than 0.001, effect size negative 1.192. Attention/vigilance: S Z 36.24 plus or minus 10.48, H C 46.91 plus or minus 9.08, statistics negative 6.269, p value less than 0.001, effect size negative 1.084. Working memory: S Z 38.46 plus or minus 14.93, H C 40.84 plus or minus 9.05, statistics negative 1.129, p value 0.261, effect size negative 0.191. Verbal learning: S Z 39.89 plus or minus 12.84, H C 46.97 plus or minus 9.27, statistics negative 3.683, p value less than 0.001, effect size negative 0.628. Visual learning: S Z 40.01 plus or minus 10.98, H C 50.34 plus or minus 6.82, statistics negative 6.599, p value less than 0.001, effect size negative 1.119. Reasoning and problem solving: S Z 39.99 plus or minus 11.33, H C 51.88 plus or minus 8.54, statistics negative 6.895, p value less than 0.001, effect size negative 1.178. Social cognition: S Z 30.46 plus or minus 8.08, H C 35.91 plus or minus 10.61, statistics negative 3.361, p value 0.001, effect size negative 0.581. FAST: S Z 46 (38.00 tilde 55.00), H C 4 (2.00 tilde 6.00), statistics negative 9.993, p value less than 0.001, effect size negative 0.860. CRASH: S Z 32.34 plus or minus 9.28, H C 41.85 plus or minus 9.81, statistics negative 5.764, p value less than 0.001, effect size negative 0.997. Most domains show significantly lower scores for S Z compared to H C, with p values less than 0.001 and negative effect sizes, except working memory, which is not significant.

Navigate back to [Table 2].

## Figure 2. Long description

Top-left panel A plots C R A S H on the x-axis from 10 to 60 and P A N S S dash N on the y-axis from 0 to 40. Data points cluster between 20 and 40 on C R A S H and 10 to 30 on P A N S S dash N. The regression line slopes downward. Correlation r equals negative 0.27, p equals 0.024. Top-right panel B plots C R A S H on the x-axis from 10 to 60 and P A N S S dash G on the y-axis from 34 to 68. Data points cluster between 20 and 40 on C R A S H and 34 to 68 on P A N S S dash G. The regression line slopes downward. Correlation r equals negative 0.31, p equals 0.009. Bottom-left panel C plots C R A S H on the x-axis from 10 to 60 and P A N S S total score on the y-axis from 60 to 120. Data points cluster between 20 and 40 on C R A S H and 70 to 110 on P A N S S total score. The regression line slopes downward. Correlation r equals negative 0.358, p equals 0.002. Bottom-right panel D plots C R A S H on the x-axis from 10 to 60 and F A S T total score on the y-axis from 0 to 12. Data points cluster between 20 and 40 on C R A S H and 2 to 10 on F A S T total score. The regression line slopes downward. Correlation r sub s equals negative 0.43, p less than 0.001.

Navigate back to Figure 2..

## [Table 3] Long description

From the top row, column headers are: Author (year), N, Age (mean plus or minus S D), Diagnostic instrument, Measure of C R, Measure of symptoms, Measure of cognitive function, Measure of functioning, Main results, Quality of the study (N O S). The first row lists Present study (2025), N equals 70, mean age 29.84 plus or minus 7.33, I C D 10, C R A S H, P A N S S, cognitive function measures include SoP: T M T-A, B A C S, C F; A V: C P T-I P; W M: W M S-III S S; verbal learning: H V L T-R; visual learning: B V M T-R; R P S: N A B Mazes; S C: M S C E I T, functioning measured by F A S T, main result is a statistically significant relationship between C R and P A N S S and F A S T, with higher C R related to better clinical symptoms and social functioning, quality 8 Good. The second row is Leeson et al. (2009), N equals 129, mean age 25.53 plus or minus 8.04, D S M-I I I R and I C D 10, premorbid I Q, P A N S S, cognitive function includes W M: spatial working memory task; verbal learning: R A V L T; R P S: Tower of London task, functioning N A, main result is both low and deteriorated groups had more core negative symptoms, verbal learning, working memory and planning correlated with premorbid I Q at P less than point zero five, quality 7 Good. The third row is Cámara et al. (2021), N equals 116, mean age 40.66 plus or minus 9.62, I C D 10, education, P A N S S, cognitive function includes SoP: symbol search; W M: digit span; R P S: phonological verbal fluency, functioning G A F, main result is education showed significant positive correlations with working memory and verbal fluency, and negative correlation with G A F, quality 8 Good. The fourth row is Correa-Ghisays et al. (2022), N equals 30, mean age 40.8 plus or minus 10.7, D S M-V, vocabulary and education, P A N S S, cognitive function includes SoP: F T T, W A I S-III digit symbol coding, S C W T color and word subtests, T M T-A; W M: T M T-B, W A I S-III digit span-B; verbal learning: T A V E C V3, V8, V10; visual learning: R O C F; R P S: S C W T color/word, W C S T-C C P E, functioning F A S T, main result is C R negatively correlated with clinical symptoms and positively with cognitive and social functioning, quality 7 good. The fifth row is Ehrminger et al. (2020), N equals 776, mean age 31.6 plus or minus 9.3, D S M-I V, premorbid I Q and education, P A N S S, cognitive function N A, functioning G A F, main result is C R scores associated with G A F and cognitive scores, quality 6 Satisfactory. The sixth row is Rodriguez et al. (2022), N equals 137, mean age 25.93 plus or minus 6.47, I C D-10, education, premorbid I Q, socioeconomic status, P A N S S, cognitive function N A, functioning G A F, main result is C R positively related to symptom severity and general functioning, quality 8 Good. The seventh row is Sánchez-Torres et al. (2023), N equals 99, mean age 26.25 plus or minus 5.78, D S M-I V, premorbid I Q, education, scholastic performance, P A N S S, cognitive function includes SoP: T M T-A; A V: C P T-I I; W M: Tower of London, W A I S-III digit span; verbal learning: A I S-III letter-number sequencing, T A V E C; visual learning: W M S-visual reproduction; S C: M S C E I T, functioning F A S T and G A F, main result is C R associated with cognitive performance in processing speed, working memory, visual memory, and social cognition, quality 7 Good. The eighth row is Amoretti et al. (2024), N equals 225, mean age 45.02 plus or minus 12.82, D S M-V, C R A S H, P A N S S, cognitive function SoP: P S T; verbal learning: V L T-D, functioning G A F, main result is C R predicted all cognitive domains, negative symptoms, and functioning, quality 9 Very Good. The ninth row is Forte et al. (2024), N equals 138, mean age 24.77 plus or minus 5.29, D S M-I V, premorbid I Q, education, lifetime participation in leisure, social, physical activities, P A N S S, cognitive function N A, functioning F A S T, main result is C R negatively correlated with clinical symptoms, quality 7 Good. The tenth row is Spinelli et al. (2025), N equals 69, mean age 39.23 plus or minus 13.85, diagnostic instrument N A, education, P A N S S, cognitive function S C: Reading the Mind in the Eyes Test, functioning N A, main result is C R plays a significant role in alleviating negative symptoms in schizophrenia, quality 7 good.

Navigate back to [Table 3].

## [Table 4] Long description

Beginning with the top row, PANSS-N reports 8 studies with 914 patients, an overall effect size r of minus 0.21, 95 percent confidence interval minus 0.31 to minus 0.10, r p-value less than 0.001, I squared 60.93 percent, Q-test 17.89, Q-test p-value 0.01. The next row, PANSS-P, includes 5 studies and 523 patients, effect size r minus 0.06, 95 percent confidence interval minus 0.16 to 0.05, r p-value 0.270, I squared 23 percent, Q-test 5.24, Q-test p-value 0.26. The final row, PANSS-G, lists 3 studies with 169 patients, effect size r minus 0.28, 95 percent confidence interval minus 0.41 to minus 0.13, r p-value less than 0.001, I squared 0.00 percent, Q-test 1.27, Q-test p-value 0.53. PANSS is defined as the Positive and Negative Syndrome Scale, PANSS-P as positive symptom, PANSS-N as negative symptom, and PANSS-G as general psychopathology symptom.

Navigate back to [Table 4].

## [Table 5] Long description

Beginning at the top row, the table lists cognitive domains in the leftmost column: Speed of processing, Working memory, Verbal learning, Visual learning, Reasoning and problem solving, and Social cognition. For Speed of processing, five studies with 540 patients yield an effect size r of 0.28, confidence interval 0.04 to 0.50, r p-value 0.020, I squared 86.87 percent, Q-test 42.16, and Q-test p-value less than 0.001. Working memory includes five studies, 444 patients, effect size r 0.25, confidence interval 0.16 to 0.34, r p-value less than 0.001, I squared 0.00 percent, Q-test 0.84, Q-test p-value 0.930. Verbal learning has five studies, 553 patients, effect size r 0.30, confidence interval 0.12 to 0.47, r p-value 0.002, I squared 77.90 percent, Q-test 17.42, Q-test p-value 0.002. Visual learning covers three studies, 199 patients, effect size r 0.33, confidence interval 0.11 to 0.52, r p-value 0.004, I squared 57.18 percent, Q-test 4.82, Q-test p-value 0.090. Reasoning and problem solving includes four studies, 345 patients, effect size r 0.31, confidence interval 0.21 to 0.40, r p-value less than 0.001, I squared 0.00 percent, Q-test 2.08, Q-test p-value 0.560. Social cognition has three studies, 238 patients, effect size r 0.08, confidence interval minus 0.19 to 0.34, r p-value 0.560, I squared 76.27 percent, Q-test 8.34, Q-test p-value 0.020. Each row presents the statistical relationship between cognitive reserve and the respective domain, with effect sizes and heterogeneity measures.

Navigate back to [Table 5].

## Figure 4. Long description

The plot has the y-axis listing, from top to bottom, Social Cognition, Reasoning and Problem Solving, Visual Learning, Verbal Learning, Working Memory, and Speed of Processing. The x-axis ranges from negative 0.3 to 0.6, labeled as the correlation coefficient. Each domain has a horizontal line representing the 95 percent confidence interval, with a square or open square marking the point estimate. Social Cognition shows a non-significant result (open square) near zero. All other domains display significant results (filled squares) with positive correlations. Verbal Learning and Working Memory have the largest effect sizes, with their squares positioned further right. A red dashed vertical line marks zero. The legend at the right identifies filled squares as significant and open squares as non-significant.

Navigate back to Figure 4..

## [Table 6] Long description

Column headers from left to right are Social function, Studies n, Patients n, Overall effect size r, 95 percent confidence interval, r p-value, I squared percent, Q-test degrees of freedom, and Q-test p-value. The first row for G A F shows 4 studies, 1254 patients, effect size r of 0.18, confidence interval 0.09 to 0.27, r p-value less than 0.001, I squared 50.21 percent, Q-test 6.08, Q-test p-value 0.110. The second row for F A S T shows 3 studies, 238 patients, effect size r of minus 0.16, confidence interval minus 0.59 to 0.34, r p-value 0.55, I squared 92.21 percent, Q-test 19.37, Q-test p-value less than 0.001. Notes clarify F A S T as Functioning Assessment Short Test and G A F as global assessment of functioning.

Navigate back to [Table 6].

## Figure 5. Long description

The horizontal axis is labeled correlation coefficient r, ranging from negative zero point four to positive zero point four. The vertical axis lists F A S T at the top and G A F below. F A S T is represented by an open square at r near zero with a wide horizontal error bar. G A F is shown by a filled square at a positive r value with a shorter error bar. A red dashed vertical line marks r equals zero. The legend at right indicates filled squares are significant, open squares are not significant.

Navigate back to Figure 5..
