# Supplementary material for: The relationship among cognitive reserve and symptoms, cognition, and functioning in schizophrenia: A case–control study and meta-analysis
Source: Eur Psychiatry. 2026 May 12;69(1):e58. doi: 10.1192/j.eurpsy.2026.12216 (PMC13247918; doi:10.1192/j.eurpsy.2026.12216)
Supplement: Hou et al. supplementary material [file S0924933826122160sup001.docx]

**Supplementary Materials**

**Index**

**1. Description of the neuropsychological battery** 3

**2. Correlations of the CRASH score with age, duration of illness, PANSS, MCCB and FAST score** 4

**3. Quality assessment using the Newcastle-Ottawa Scale (NOS)**5

**4.Forest plots, sensitivity analyses and meta-regressions**6

Correlation between cognitive reserve and positive symptoms 6

Correlation between cognitive reserve and positive symptoms, forest plot 6

Correlation between cognitive reserve and negative symptoms 7

Correlation between cognitive reserve and negative symptoms, forest plot 7

Correlation between cognitive reserve and negative symptoms, sensitivity analysis 7

Correlation between cognitive reserve and negative symptoms, meta-regression 7

Correlation between cognitive reserve and general psychopathology symptoms 8

Correlation between cognitive reserve and general psychopathology symptoms, forest plot 8

Correlation between cognitive reserve and speed of processing 9

Correlation between cognitive reserve and speed of processing, forest plot 9

Correlation between cognitive reserve and speed of processing, sensitivity analysis 9

Correlation between cognitive reserve and speed of processing, meta-regression9

Correlation between cognitive reserve and working memory 10

Correlation between cognitive reserve and working memory, forest plot 10

Correlation between cognitive reserve and verbal learning 11

Correlation between cognitive reserve and verbal learning, forest plot 11

Correlation between cognitive reserve and s verbal learning, sensitivity analysis 11

Correlation between cognitive reserve and verbal learning, meta-regression 11

Correlation between cognitive reserve and visual learning 12

Correlation between cognitive reserve and visual learning, forest plot 12

Correlation between cognitive reserve and visual learning, sensitivity analysis12

Correlation between cognitive reserve and visual learning, meta-regression 12

Correlation between cognitive reserve and reasoning and problem solving 13

Correlation between cognitive reserve and reasoning and problem solving, forest plot 13

Correlation between cognitive reserve and social cognition 14

Correlation between cognitive reserve and social cognition, forest plot 14

Correlation between cognitive reserve and social cognition, sensitivity analysis14

Correlation between cognitive reserve and social cognition, meta-regression 14

Correlation between cognitive reserve and FAST 15

Correlation between cognitive reserve and FAST, forest plot 15

Correlation between cognitive reserve and FAST, sensitivity analysis 15

Correlation between cognitive reserve and FAST, meta-regression 15

Correlation between cognitive reserve and GAF 16

Correlation between cognitive reserve and GAF, forest plot 16

Correlation between cognitive reserve and GAF, sensitivity analysis 16

Correlation between cognitive reserve and GAF, meta-regression 16

**1. Description of the neuropsychological battery**

| Cognitive domain | Subtests used |
| --- | --- |
| Speed of Processing (SoP) | Trail Making Test A (TMT-A), Brief Assessment of Cognition in Schizophrenia (BACS), Category Fluency (CF) |
| Attention/Vigilance (AV) | Continuous Performance Test-Identical Paris (CPT-IP) |
| Working Memory (WM) | Wechsler Memory Scale-Third Edition：Spatial Span (WMS-III SS) |
| Verbal Learning (Vrbl Lrng) | Hopkins Verbal Learning Test-Revised (HVLT-R) |
| Visual Learning (Vis Lrng) | Brief Visuospatial Memory Test Revised (BVMT-R） |
| Reasoning and Problem Solving (RPS) | Neuropsychological Assessment Battery：Mazes (NAB Mazes) |
| Social Cognition (SC) | Mayer-Salovey-Caruso Emotion Intelligence Test (MSCEIT) |

**2. Correlations of the CRASH score with age, duration of illness, PANSS, MCCB and FAST score**

|  | r | p | lowerCI | upperCI |
| --- | --- | --- | --- | --- |
| **SZ group** |  |  |  |  |
| Age | -0.018 | 0.882 | -0.252 | 0.218 |
| Duration of illness (years) | -0.016 | 0.893 | -0.250 | 0.220 |
| PANSS total score | -0.358 | 0.002 | -0.547 | -0.134 |
| PANSS-P | -0.064 | 0.599 | -0.295 | 0.174 |
| PANSS-N | -0.27 | 0.024 | -0.475 | -0.038 |
| PANSS-G | -0.31 | 0.009 | -0.508 | -0.08 |
| Speed of Processing | 0.012 | 0.919 | -0.223 | 0.247 |
| Attention/Vigilance | -0.004 | 0.977 | -0.238 | 0.232 |
| Working Memory | 0.203 | 0.092 | -0.033 | 0.418 |
| Verbal Learning | 0.038 | 0.757 | -0.199 | 0.27 |
| Visual Learning | 0.181 | 0.135 | -0.057 | 0.399 |
| Reasoning and Problem Solving | 0.21 | 0.081 | -0.026 | 0.424 |
| Social Cognition | -0.195 | 0.106 | -0.411 | 0.042 |
| FAST | -0.547 | <0.001 | -0.697 | -0.352 |
| **HC group** |  |  |  |  |
| Speed of Processing | 0.126 | 0.321 | -0.124 | 0.361 |
| Attention/Vigilance | -0.151 | 0.233 | -0.383 | 0.098 |
| Working Memory | 0.117 | 0.356 | -0.132 | 0.353 |
| Verbal Learning | -0.130 | 0.306 | -0.364 | 0.120 |
| Visual Learning | 0.124 | 0.331 | -0.126 | 0.358 |
| Reasoning and Problem Solving | 0.016 | 0.900 | -0.231 | 0.261 |
| Social Cognition | 0.093 | 0.466 | -0.157 | 0.331 |
| FAST | -0.540 | <0.001 | -0.694 | -0.339 |

**3. Quality assessment using the Newcastle-Ottawa Scale (NOS)**

| Auther, Year | Representativeness of the sample 1) | Sample Size 2) | Measurement  of CR 3) | Measurement of symptoms, cognition, and functioning 4) | Confounding factors  Controlled 1) | Assessment of  Outcome 1) | Statistical test 2) | Overall evaluation |
| --- | --- | --- | --- | --- | --- | --- | --- | --- |
| Present study, 2025 | * |  | ** | ** |  | ** | * | 8 Good |
| VerityC.Leeson, 2009 | * | * | * | ** |  | ** |  | 7 Good |
| Silvia Cámara, 2021 | * | * | * | ** |  | ** | * | 8 Good |
| Patricia Correa-Ghisays, 2022 | * |  | * | ** |  | ** | * | 7 good |
| Mickael Ehrminger, 2020 | * | * |  | ** |  | ** |  | 6 Satisfactory |
| Mabel Rodriguez, 2022 | * | * | * | ** |  | ** | * | 8 Good |
| Ana M. Sánchez-Torres, 2023 | * |  |  | ** | * | ** | * | 7 Good |
| Silvia Amoretti, 2024 | * | * | ** | ** |  | ** | * | 9 Very Good |
| M. Florencia Forte, 2024 | * | * | * | ** |  | ** |  | 7 Good |
| Sebasti´an Lema Spinelli, 2025 | * |  | * | ** |  | ** | * | 7 good |

**4.Forest plots, sensitivity analyses and meta-regressions**

Correlation between cognitive reserve and positive symptoms

*Correlation between cognitive reserve and positive symptoms, forest plot*


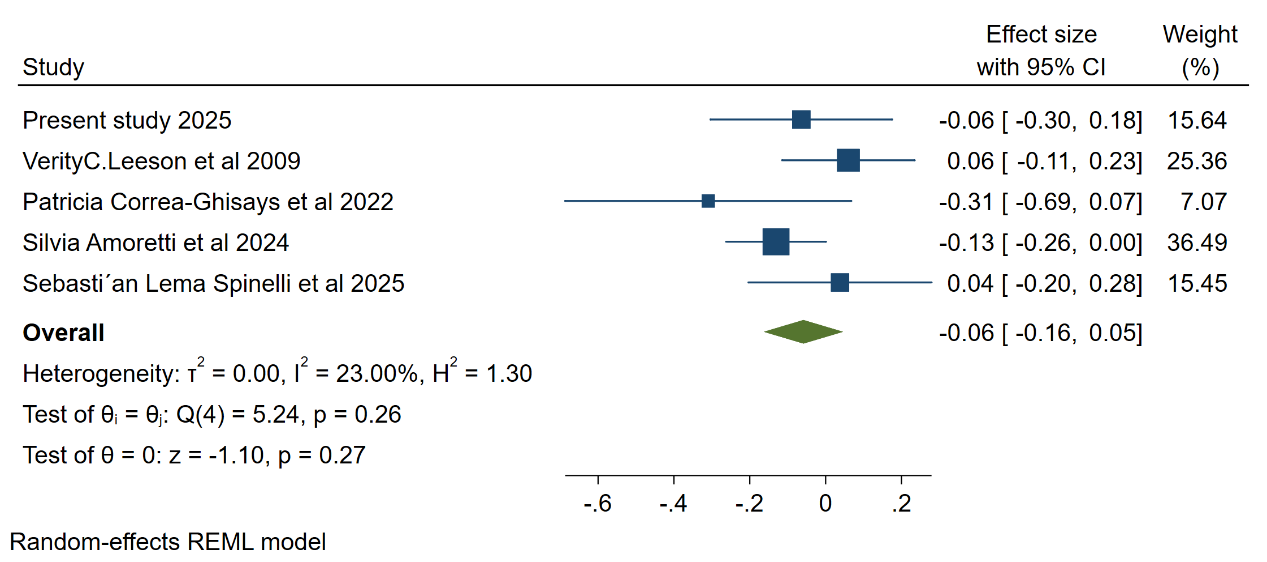


Correlation between cognitive reserve and negative symptoms

*Correlation between cognitive reserve and negative symptoms, forest plot*


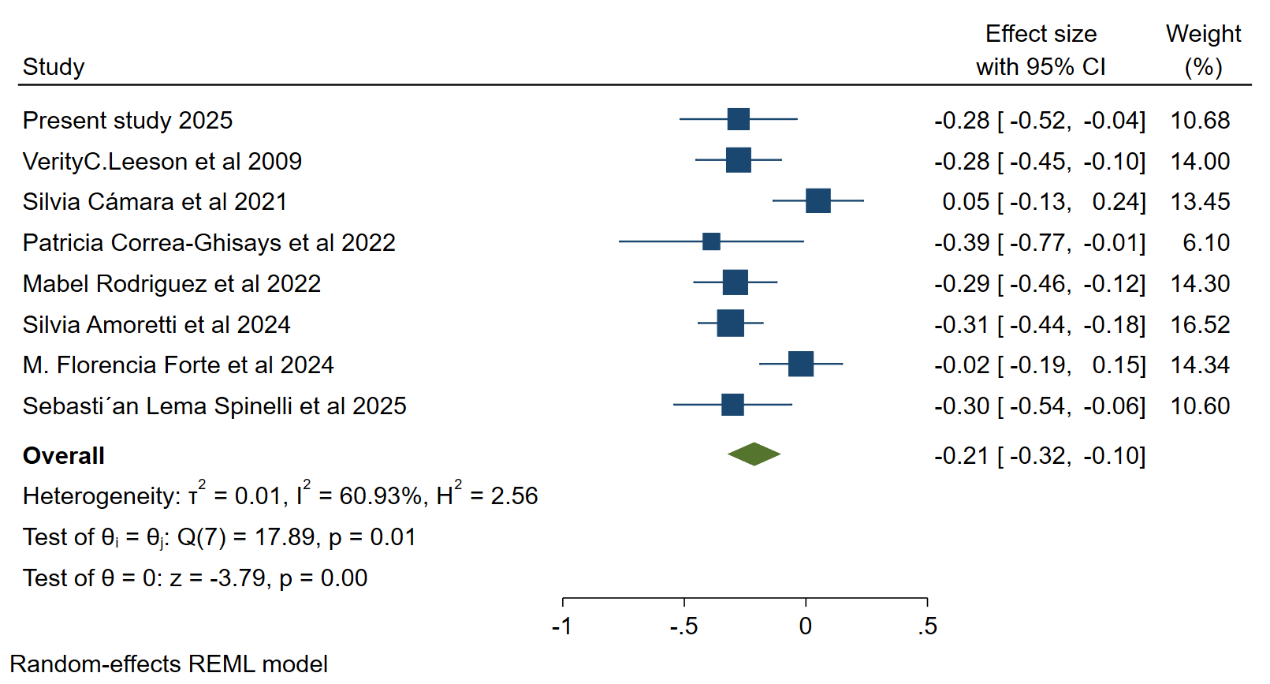


*Correlation between cognitive reserve and negative symptoms, sensitivity analysis*

| Study omitted | Sample (n) | Effect size, r | 95% CI | r p-value | Comparison with main analysis |
| --- | --- | --- | --- | --- | --- |
| None | 914 | -0.21 | -0.31, -0.10 | ＜0.01 | Reference |
| Present study, 2025 | 844 | -0.20 | -0.32 ~ -0.08 | ＜0.01 | Effect slightly attenuated, CI widened |
| VerityC.Leeson et al, 2009 | 785 | -0.20 | -0.32 ~ -0.08 | ＜0.01 | Effect slightly attenuated, CI widened |
| Silvia Cámara et al, 2021 | 798 | -0.25 | -0.33 ~ -0.16 | ＜0.01 | Effect strengthened, CI narrowed |
| Patricia Correa-Ghisays et al, 2022 | 884 | -0.20 | -0.31 ~ -0.09 | ＜0.01 | Effect slightly attenuated, CI similar |
| Mabel Rodriguez et al, 2022 | 777 | -0.20 | -0.32 ~ -0.08 | ＜0.01 | Effect slightly attenuated, CI widened |
| Silvia Amoretti et al, 2024 | 689 | -0.19 | -0.20 ~ -0.07 | ＜0.01 | Effect slightly attenuated, CI narrowed substantially |
| M. Florencia Forte et al, 2024 | 776 | -0.24 | -0.34 ~ -0.14 | ＜0.01 | Effect strengthened, CI similar |
| Sebasti´an Lema Spinelli et al, 2025 | 845 | -0.20 | -0.31 ~ -0.08 | ＜0.01 | Effect slightly attenuated, CI widened |
| Range | 689~ 914 | -0.19 ~ -0.25 | -0.20 ~ -0.16 | <0.01 | All analyses remain statistically significant |

*Correlation between cognitive reserve and negative symptoms, meta-regression*

|  | Effect size | SE | P | 95%CI |
| --- | --- | --- | --- | --- |
| age |  |  |  |  |
| 8 | -0.002 | 0.008 | 0.80 | -0.017~0.013 |
| % of females |  |  |  |  |
| 8 | -0.005 | 0.007 | 0.48 | -0.018~0.009 |
| duration of illness |  |  |  |  |
| 6 | 0.013 | 0.016 | 0.431 | -0.019~0.044 |
| age at onset |  |  |  |  |
| 6 | 0.005 | 0.022 | 0.824 | -0.038~0.048 |

Correlation between cognitive reserve and general psychopathology symptoms

*Correlation between cognitive reserve and general psychopathology symptoms, forest plot*


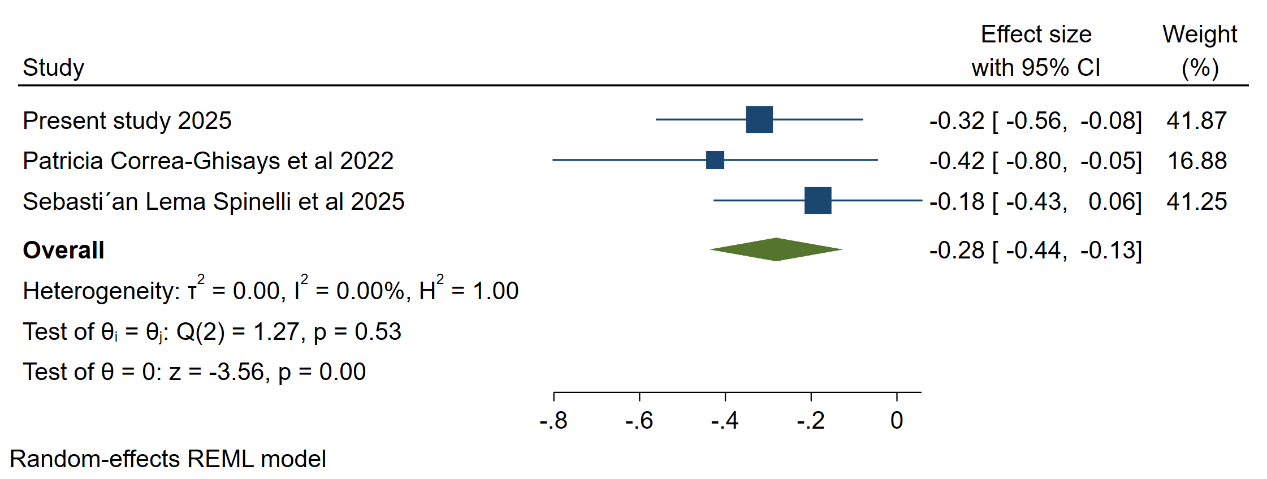


Correlation between cognitive reserve and speed of processing

*Correlation between cognitive reserve and speed of processing, forest plot*


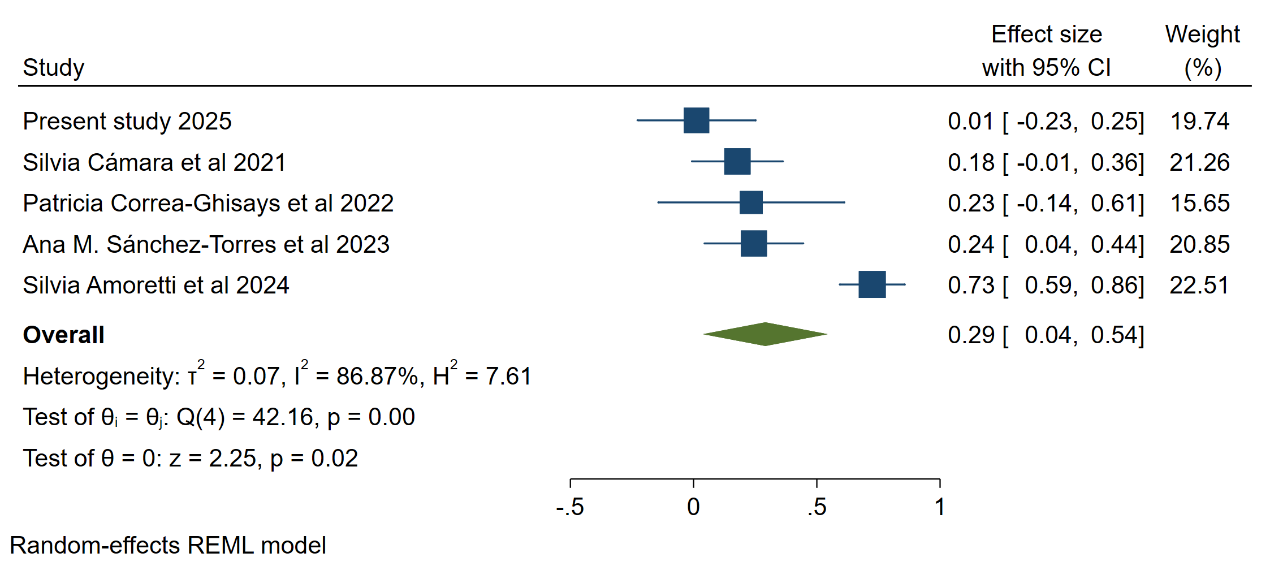


*Correlation between cognitive reserve and speed of processing, sensitivity analysis*

| Study omitted | Sample (n) | Effect size, r | 95% CI | r p-value | Comparison with main analysis |
| --- | --- | --- | --- | --- | --- |
| None | 540 | 0.28 | 0.04 ~ 0.5 | 0.02 | Reference |
| Present study, 2025 | 470 | 0.35 | 0.089 ~ 0.56 | ＜0.01 | Effect strengthened, significance improved |
| Silvia Cámara et al, 2021 | 424 | 0.31 | 0.002 ~ 0.56 | 0.05 | Effect slightly strengthened, CI widened, significance marginal |
| Patricia Correa-Ghisays et al, 2022 | 510 | 0.29 | -0.01 ~ 0.54 | 0.06 | Effect similar, CI widened, significance lost |
| Ana M. Sánchez-Torres et al, 2023 | 441 | 0.30 | 0.06 ~ 0.55 | 0.07 | Effect slightly strengthened, significance marginal |
| Silvia Amoretti et al, 2024 | 315 | 0.17 | 0.06 ~ 0.27 | ＜0.01 | Effect substantially attenuated, CI narrowed considerably |
| Range | 315 ~540 | 0.17 ~ 0.35 | -0.01 ~ 0.56 | 0.06 ~ <0.01 | Moderate sensitivity to individual study exclusion |

*Correlation between cognitive reserve and speed of processing, meta-regression*

|  | Effect size | SE | P | 95%CI |
| --- | --- | --- | --- | --- |
| age |  |  |  |  |
| 5 | 0.02 | 0.02 | 0.16 | -0.01~0.05 |
| % of females |  |  |  |  |
| 5 | -0.005 | 0.01 | 0.72 | -0.03~0.02 |
| positive symptoms |  |  |  |  |
| 5 | -0.02 | 0.03 | 0.39 | -0.08~0.03 |
| negative symptoms |  |  |  |  |
| 5 | 0.002 | 0.05 | 0.97 | -0.09~0.09 |
| duration of illness |  |  |  |  |
| 4 | 0.032 | 0.032 | 0.323 | -0.031~0.095 |
| age at onset |  |  |  |  |
| 3 | -0.038 | 0.043 | 0.375 | -0.122~0.046 |

Correlation between cognitive reserve and working memory

*Correlation between cognitive reserve and working memory, forest plot*


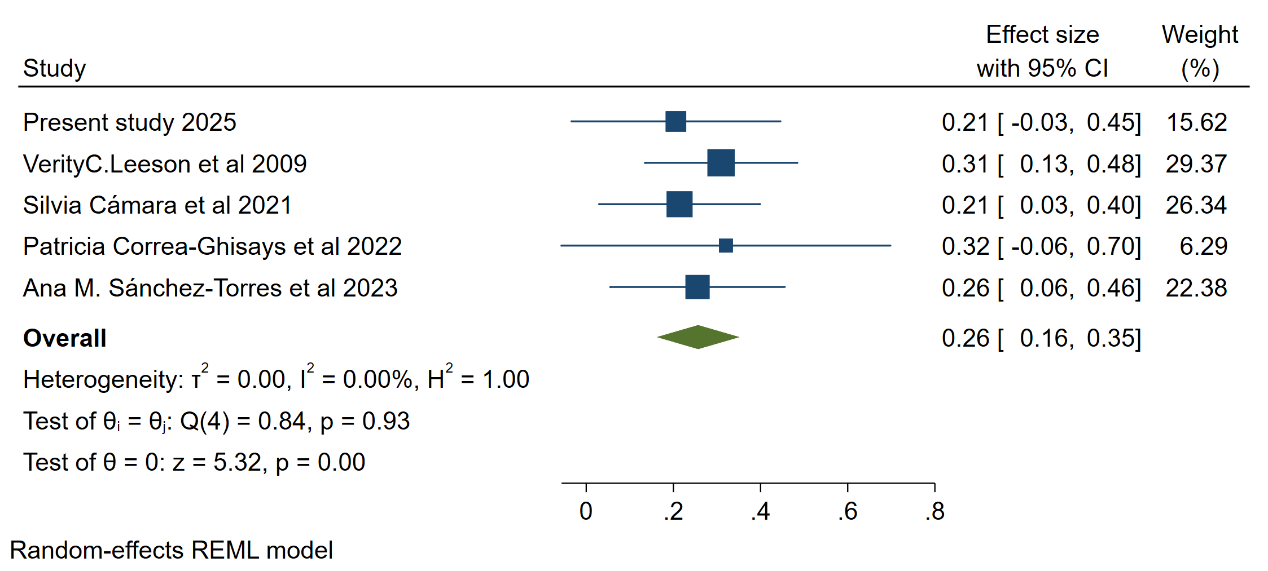


Correlation between cognitive reserve and verbal learning

*Correlation between cognitive reserve and verbal learning, forest plot*


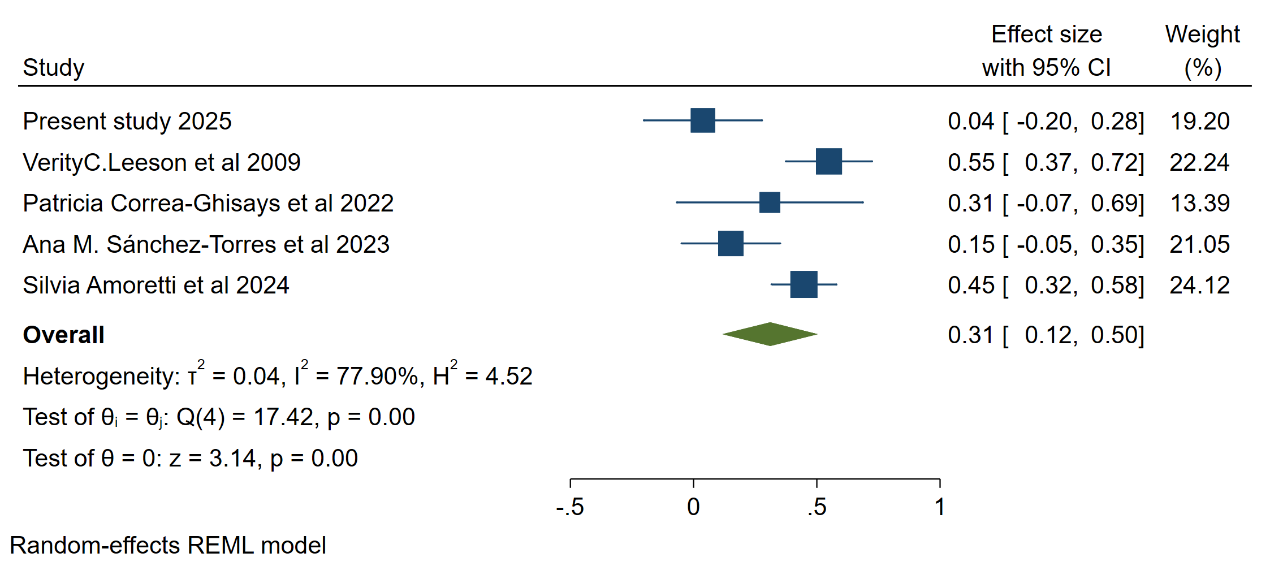


*Correlation between cognitive reserve and s verbal learning, sensitivity analysis*

| Study omitted | Sample (n) | Effect size,r | 95% CI | r p-value | Comparison with main analysis |
| --- | --- | --- | --- | --- | --- |
| None | 553 | 0.30 | 0.12 ~ 0.47 | ＜0.01 | Reference |
| Present study 2025 | 483 | 0.36 | 0.19 ~ 0.51 | ＜0.01 | Effect strengthened, CI narrowed |
| VerityC.Leeson et al 2009 | 424 | 0.24 | 0.05 ~ 0.42 | 0.02 | Effect attenuated, significance reduced |
| Patricia Correa-Ghisays et al 2022 | 523 | 0.30 | 0.08 ~ 0.49 | ＜0.01 | Effect unchanged, CI widened |
| Ana M. Sánchez-Torres et al 2023 | 454 | 0.34 | 0.13 ~ 0.52 | ＜0.01 | Effect strengthened, CI similar |
| Silvia Amoretti et al 2024 | 328 | 0.26 | 0.03 ~ 0.47 | 0.03 | Effect attenuated, significance reduced |
| Range | 328 ~ 553 | 0.24 ~ 0.36 | 0.03 ~ 0.52 | 0.02 ~ <0.01 | Good robustness, all analyses remain significant |

*Correlation between cognitive reserve and verbal learning, meta-regression*

|  | effect size | SE | P | 95%CI |
| --- | --- | --- | --- | --- |
| age |  |  |  |  |
| 5 | 0.006 | 0.01 | 0.67 | -0.02~0.03 |
| % of females |  |  |  |  |
| 5 | -0.002 | 0.01 | 0.82 | -0.02~0.02 |
| duration of illness |  |  |  |  |
| 3 | 0.035 | 0.012 | 0.004 | 0.011~0.058 |
| age at onset |  |  |  |  |
| 3 | -0.295 | 0.087 | 0.001 | -0.466~-0.125 |
| negative symptoms |  |  |  |  |
| 4 | -0.015 | 0.020 | 0.437 | -0.053~0.023 |
| positive symptoms |  |  |  |  |
| 4 | -0.020 | 0.041 | 0.623 | -0.060~0.100 |
| general psychopathology symptoms |  |  |  |  |
| 4 | -0.010 | 0.016 | 0.528 | -0.041~0.021 |

Correlation between cognitive reserve and visual learning

*Correlation between cognitive reserve and visual learning, forest plot*
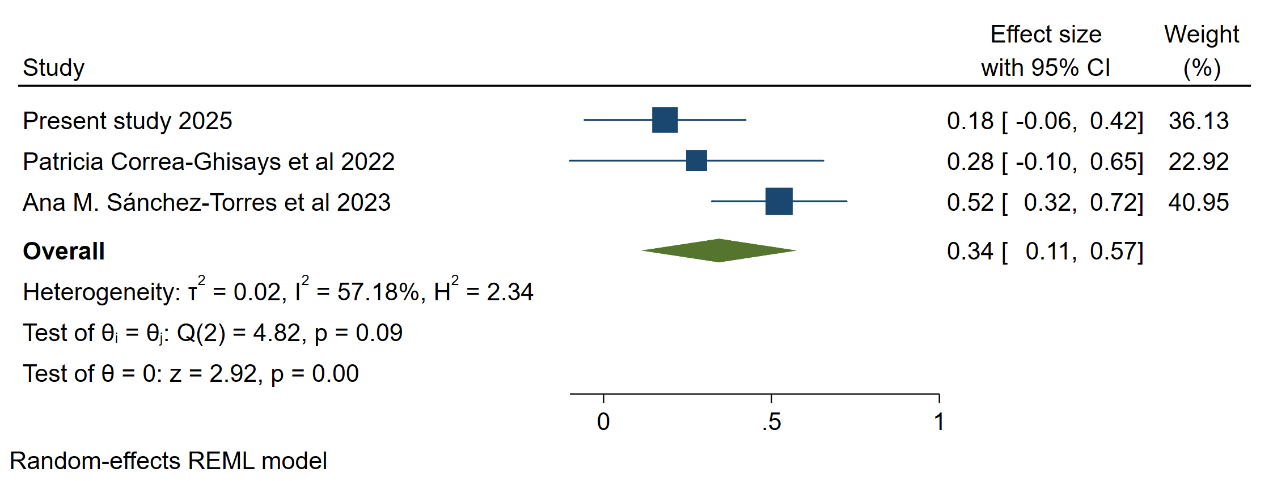


*Correlation between cognitive reserve and visual learning, sensitivity analysis*

| Study omitted | Sample (n) | Effect size,r | 95% CI | r p-value | Comparison with main analysis |
| --- | --- | --- | --- | --- | --- |
| None | 199 | 0.33 | 0.11 ~ 0.52 | ＜0.01 | Reference |
| Present study 2025 | 129 | 0.43 | 0.23 ~ 0.59 | ＜0.01 | Effect substantially strengthened, CI narrowed |
| Patricia Correa-Ghisays et al, 2022 | 169 | 0.35 | 0.03 ~ 0.60 | 0.03 | Effect slightly strengthened, CI widened substantially |
| Ana M. Sánchez-Torres et al, 2023 | 100 | 0.21 | 0.01 ~ 0.39 | 0.04 | Effect substantially attenuated, CI narrowed |
| Range | 100 ~ 199 | 0.21 ~ 0.43 | 0.01 ~ 0.60 | 0.04 ~ <0.01 | High sensitivity |

*Correlation between cognitive reserve and visual learning, meta-regression*

|  | Effect size | SE | P | 95%CI |
| --- | --- | --- | --- | --- |
| age |  |  |  |  |
| 3 | -0.01 | 0.02 | 0.57 | -0.06~0.03 |
| % of females |  |  |  |  |
| 3 | -0.008 | 0.008 | 0.345 | -0.02~0.008 |
| positive symptoms |  |  |  |  |
| 3 | -0.02 | 0.01 | 0.054 | -0.04~0.0003 |
| negative symptoms |  |  |  |  |
| 3 | -0.05 | 0.02 | 0.03 | -0.10~-0.01 |
| general psychopathology symptoms |  |  |  |  |
| 3 | -0.018 | 0.008 | 0.03 | -0.034~-0.001 |

Correlation between cognitive reserve and reasoning and problem solving

*Correlation between cognitive reserve and reasoning and problem solving, forest plot*


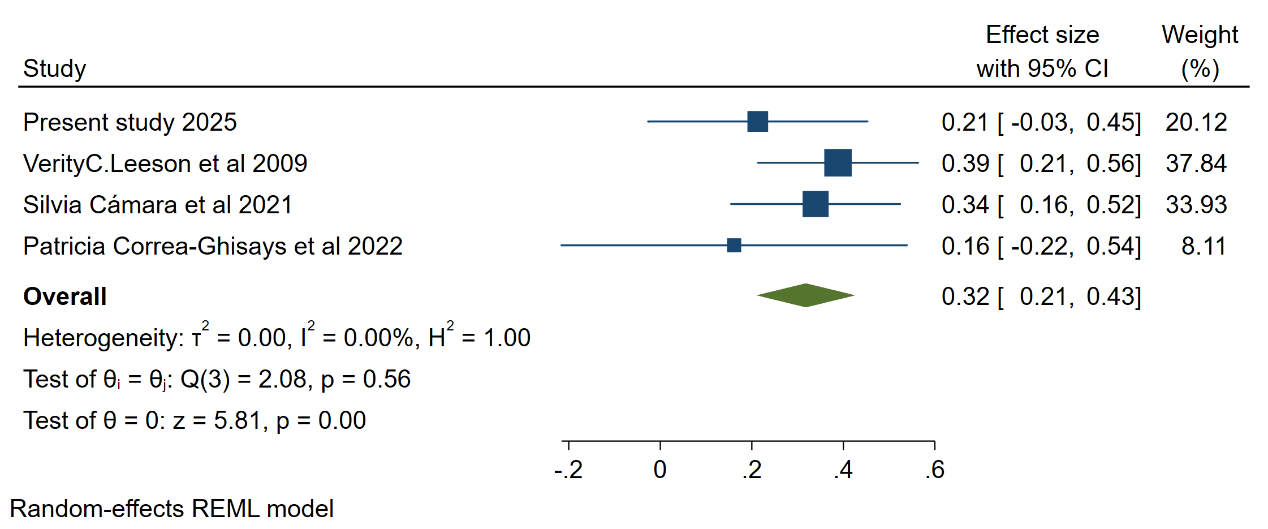


Correlation between cognitive reserve and social cognition

*Correlation between cognitive reserve and social cognition, forest plot*


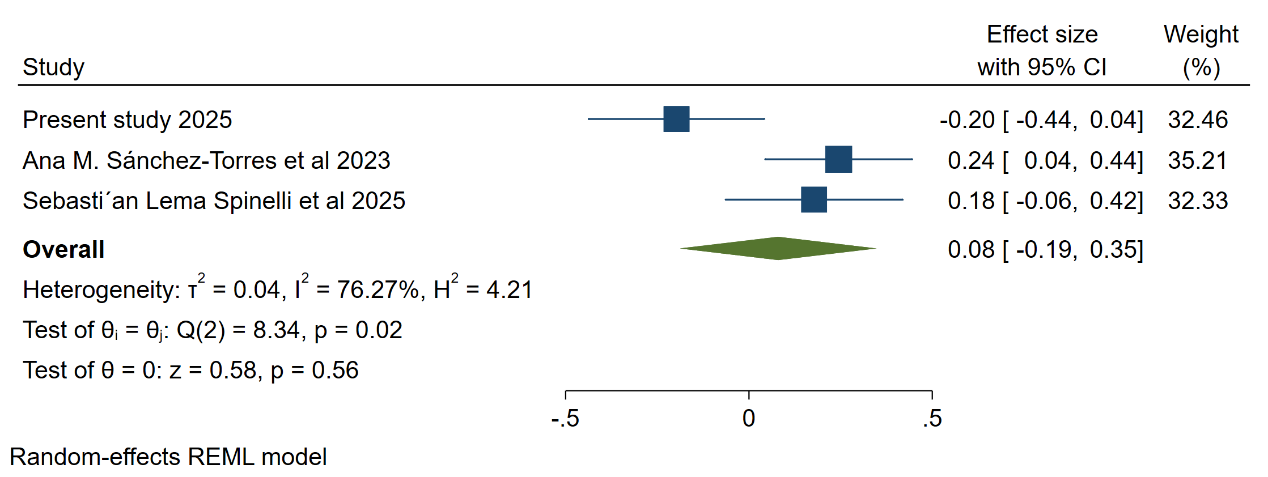
*Correlation between cognitive reserve and social cognition, sensitivity analysis*

| Study omitted | Sample (n) | Effect size,r | 95% CI | r p-value | Comparison with main analysis |
| --- | --- | --- | --- | --- | --- |
| None | 238 | 0.08 | -0.19 ~ 0.34 | 0.56 | Reference |
| Present study 2025 | 168 | 0.22 | 0.06 ~ 0.36 | ＜0.01 | Effect direction reversed, became statistically significant |
| Ana M. Sánchez-Torres et al 2023 | 139 | -0.01 | -0.36 ~ 0.34 | 0.96 | Effect direction reversed and attenuated, remained non-significant |
| Sebasti´an Lema Spinelli et al 2025 | 169 | 0.03 | -0.38 ~ 0.43 | 0.90 | Effect attenuated, remained non-significant |
| Range | 139 ~ 238 | -0.01 ~ 0.22 | -0.38 ~ 0.43 | 0.56 ~ <0.01 | Critical sensitivity |

*Correlation between cognitive reserve and social cognition, meta-regression*

|  | Effect size | SE | P | 95%CI |
| --- | --- | --- | --- | --- |
| % of females |  |  |  |  |
| 3 | 0.003 | 0.035 | 0.92 | -0.07~0.07 |
| sex |  |  |  |  |
| 3 | -0.01 | 0.008 | -0.08 | -0.03~0.002 |
| positive symptoms |  |  |  |  |
| 3 | -0.03 | 0.01 | 0.005 | -0.05~-0.01 |
| negative symptoms |  |  |  |  |
| 3 | -0.029 | 0.05 | 0.55 | -0.13~0.07 |
| general psychopathology symptoms |  |  |  |  |
| 3 | -0.02 | 0.008 | 0.004 | -0.04~-0.007 |

Correlation between cognitive reserve and FAST

*Correlation between cognitive reserve and FAST, forest plot
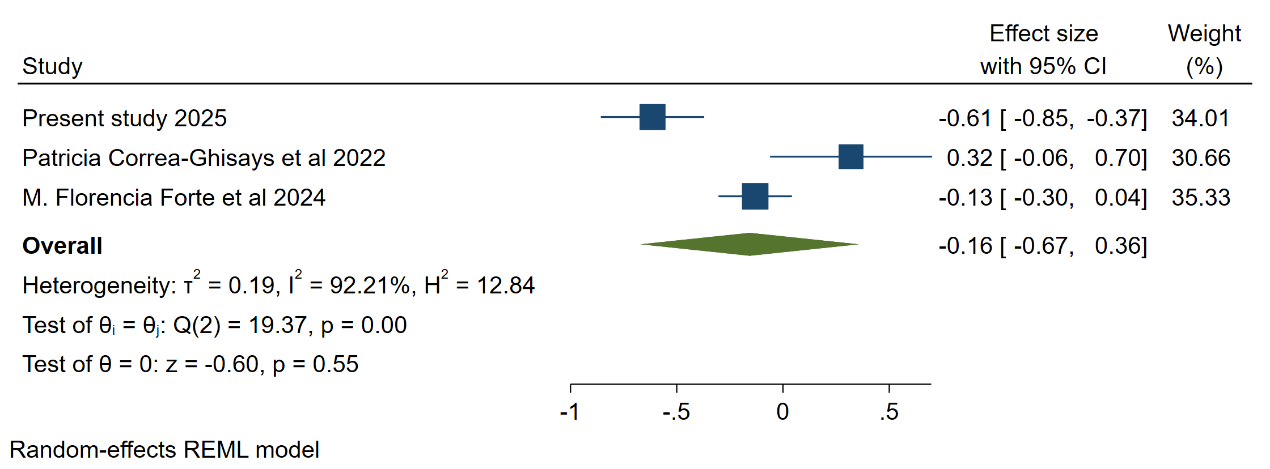
*

*Correlation between cognitive reserve and FAST, sensitivity analysis*

| Study omitted | Sample (n) | Effect size,r | 95% CI | r p-value | Comparison with main analysis |
| --- | --- | --- | --- | --- | --- |
| None | 238 | -0.16 | -0.59 ~ 0.34 | 0.55 | Reference |
| Present study, 2025 | 168 | 0.06 | -0.36 ~ 0.46 | 0.78 | Effect direction reversed, remained non-significant |
| Patricia Correa-Ghisays et al, 2022 | 208 | -0.35 | -0.69 ~ 0.11 | 0.13 | Effect strengthened (more negative), significance improved |
| M. Florencia Forte et al, 2024 | 100 | -0.16 | -0.79 ~ 0.64 | 0.73 | Effect unchanged, CI widened substantially |
| Range | 100 ~ 238 | -0.35 ~ 0.06 | -0.79 ~ 0.64 | 0.13 ~ 0.78 | Critical instability and lack of robustness |

*Correlation between cognitive reserve and FAST, meta-regression*

|  | Effect size | SE | P | 95%CI |
| --- | --- | --- | --- | --- |
| age |  |  |  |  |
| 3 | 0.04 | 0.04 | 0.41 | -0.05~0.12 |
| % of females |  |  |  |  |
| 3 | -0.02 | 0.008 | 0.003 | -0.04~-0.009 |
| age at onset of SZ |  |  |  |  |
| 3 | -0.32 | 0.45 | 0.48 | -1.22~0.56 |

Correlation between cognitive reserve and GAF

*Correlation between cognitive reserve and GAF, forest plot*


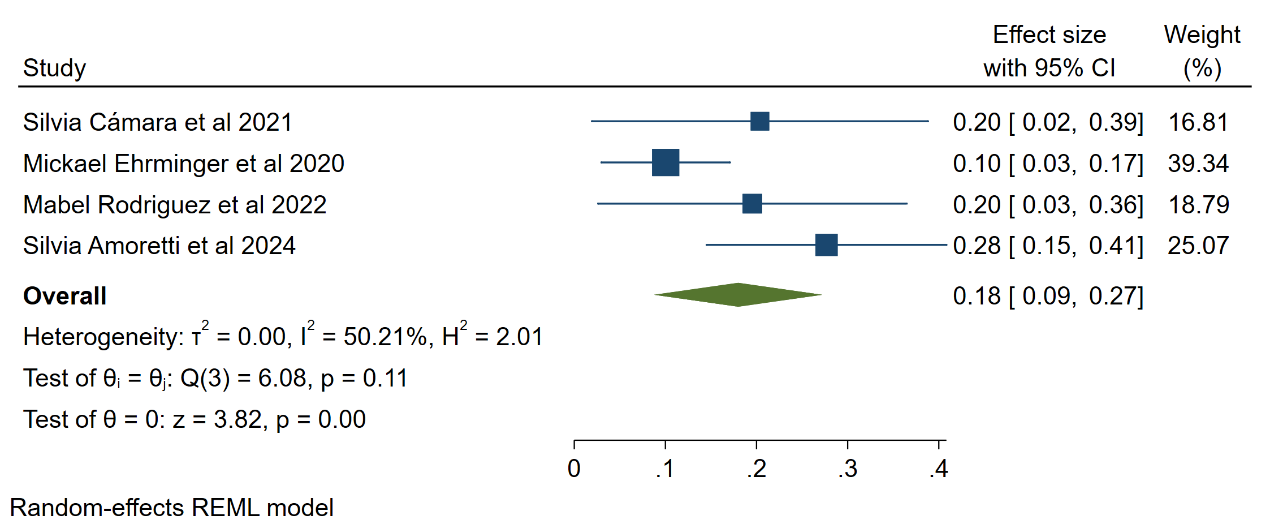


*Correlation between cognitive reserve and GAF, sensitivity analysis*

| Study omitted | Sample (n) | Effect size,r | 95% CI | r p-value | Comparison with main analysis |
| --- | --- | --- | --- | --- | --- |
| None | 1254 | 0.18 | 0.09 ~ 0.27 | ＜0.01 | Reference |
| Silvia Cámara et al, 2021 | 1138 | 0.18 | 0.07 ~ 0.28 | ＜0.01 | Effect unchanged, CI slightly widened |
| Mickael Ehrminger et al, 2020 | 478 | 0.23 | 0.14 ~ 0.32 | ＜0.01 | Effect strengthened, CI narrowed |
| Mabel Rodriguez et al, 2022 | 1117 | 0.18 | 0.06 ~ 0.29 | ＜0.01 | Effect unchanged, CI slightly widened |
| Silvia Amoretti et al, 2024 | 1029 | 0.13 | 0.06 ~ 0.20 | ＜0.01 | Effect attenuated, CI narrowed |
| Range | 478 ~ 1254 | 0.13 ~ 0.23 | 0.06 ~ 0.32 | <0.01 | Excellent robustness |

*Correlation between cognitive reserve and GAF, meta-regression*

|  | Effect size | SE | P | 95%CI |
| --- | --- | --- | --- | --- |
| % of females |  |  |  |  |
| 4 | 0.01 | 0.004 | 0.015 | 0.002~0.018 |
| positive symptoms |  |  |  |  |
| 4 | -0.022 | 0.028 | 0.43 | -0.08~0.03 |
| negative symptoms |  |  |  |  |
| 4 | -0.005 | 0.02 | 0.847 | -0.05~0.044 |
| general psychopathology symptoms |  |  |  |  |
| 3 | -0.022 | 0.011 | 0.046 | -0.043~-0.0004 |
